# Indoxyl sulfate- and P-cresol-induced monocyte adhesion and migration is mediated by integrin-linked kinase-dependent podosome formation

**DOI:** 10.1038/s12276-022-00738-8

**Published:** 2022-03-04

**Authors:** Sofía Campillo, Lourdes Bohorquez, Elena Gutiérrez-Calabrés, Diego García-Ayuso, Verónica Miguel, Mercedes Griera, Yolanda Calle, Sergio de Frutos, Manuel Rodríguez-Puyol, Diego Rodríguez-Puyol, Laura Calleros

**Affiliations:** 1 Department of Systems Biology, Physiology Unit, Universidad de Alcalá, Alcalá de Henares, Spain; 2 Instituto Ramón y Cajal de Investigación Sanitaria (IRYCIS), Fundación Renal Iñigo Álvarez de Toledo (FRIAT) and REDinREN, (ISCIII), Madrid, Spain; 3 Program of Physiological and Pathological Processes, Centro de Biología Molecular “Severo Ochoa“, (CSIC-UAM), Madrid, Spain; 4 Department of Life Sciences, University of Roehampton, London, UK; 5 Department of Medicine and Medical Specialties, Universidad de Alcalá, Alcalá de Henares, Spain; 6 Biomedical Research Foundation and Nephrology Unit, Hospital Universitario Príncipe de Asturias, Alcalá de Henares, Spain

**Keywords:** Experimental models of disease, Atherosclerosis, Mechanisms of disease, Integrins, End-stage renal disease

## Abstract

Cardiovascular disease is an important cause of death in patients with chronic kidney disease (CKD). Protein-bound uremic toxins, such as *p*-cresyl and indoxyl sulfate (IS), are poorly removed during hemodialysis, leading to vascular endothelial dysfunction and leukocyte extravasation. These processes can be related to dynamic adhesion structures called podosomes. Several studies have indicated the role of integrin-linked kinase (ILK) in the accumulation of integrin-associated proteins in podosomes. Here, we investigated the involvement of ILK and podosome formation in the adhesion and extravasation of monocytes under *p*-cresol (pc) and IS exposure. Incubation of THP-1 human monocyte cells with these toxins upregulated ILK kinase activity. Together, both toxins increased cell adhesion, podosome formation, extracellular matrix degradation, and migration of THP-1 cells, whereas ILK depletion with specific small interfering RNAs suppressed these processes. Interestingly, F-actin colocalized with cortactin in podosome cores, while ILK was colocalized in podosome rings under toxin stimulation. Podosome Wiskott-Aldrich syndrome protein (WASP)-interacting protein (WIP) and AKT protein depletion demonstrated that monocyte adhesion depends on podosome formation and that the ILK/AKT signaling pathway is involved in these processes. Ex vivo experiments showed that both toxins induced adhesion and podosome formation in leukocytes from wild-type mice, whereas these effects were not observed in leukocytes of conditional ILK-knockdown animals. In summary, under pc and IS stimulation, monocytes increase podosome formation and transmigratory capacity through an ILK/AKT signaling pathway-dependent mechanism, which could lead to vascular injury. Therefore, ILK could be a potential therapeutic target for the treatment of vascular damage associated with CKD.

## Kidney disease: Enzyme linked to cardiovascular complications

An enzyme involved in organizing structural proteins into protrusions of the cell membrane helps facilitate the movement of white blood cells that occurs in chronic kidney disease and can lead to cardiovascular damage. Laura Calleros from the University of Alcalá, Madrid, Spain, and colleagues showed how toxic metabolites that build up in the bloodstream as kidneys fail induce the activity of an enzyme called integrin-linked kinase (ILK). This enzyme in turn spurs white blood cells to form protrusions called podosomes that make the cells more mobile, enabling them to move out of blood vessels and into surrounding tissues where they can cause inflammation -mediated injury. ILK inactivation reversed these effects. Therapies that target the enzyme could therefore help limit the cardiovascular complications of chronic kidney disease.

## Introduction

Chronic kidney disease (CKD) is a global health problem of substantial importance due to its high prevalence and its association with an increased risk for cardiovascular disease (CVD), a major cause of death in this population^1,2^. Vascular damage is initiated by endothelial dysfunction and monocyte activation. The physiological functions of monocytes include their roles in innate immune system homeostasis, immune defense, and tissue repair, and they are implicated in the development of atherosclerosis^3^. Dialyzed and nondialyzed patients with CKD have abnormally high proportions of intermediate (CD14++/CD16+) monocytes, which have important proinflammatory and atherogenic features^4,5^ and are associated with atherosclerotic disease^6^ and cardiovascular events^7^.

In patients with advanced CKD, elevated serum concentrations of several circulating uremic toxins, notably indoxyl sulfate (IS) and *p*-cresyl sulfate (pCS), correlate with inflammatory markers^8^. Furthermore, increased levels of pCS are associated with cardiovascular complications and mortality in both CKD patients undergoing dialysis and those not undergoing dialysis^9^, whereas elevated levels of IS are associated with increased mortality in CKD patients but not with an increased risk of cardiovascular mortality^10,11^. A compelling body of evidence suggests that uremic toxins may predispose patients to CVD through increased monocyte adhesion, rolling, and extravasation. In patients with CKD, intermediate monocyte subtypes express a proatherogenic pattern of chemokines and adhesion molecules and strengthened adhesion to endothelial cell monolayers^12^. In vitro, IS increases the adhesion of THP-1 monocytes to activated human endothelial cells^13^, and pCS has been reported to induce increased oxidative burst activity of monocytes^14^. In cultured endothelial cells and macrophages, pCS promotes the expression of inflammatory factors and adhesion molecules via reactive oxygen species (ROS) production, an effect also reported in leukocyte–endothelium interactions in vivo^15^. Furthermore, intravital microscopy of rat peritoneal capillary venules after superfusion with a solution containing high concentrations of pCS and IS, among other toxins, has revealed an increase in the number of rolling leukocytes along the vascular endothelium^16^. Administration of IS to rats^16^ or mice with normal^17^ or impaired^18^ renal function also induces leukocyte adherence to the vessel wall and enhances leukocyte extravasation^16^. Overall, this evidence suggests that uremic toxins favor monocyte extravasation and subsequent inflammation-induced CVD, but the underlying mechanisms are not completely defined.

Podosomes are highly dynamic adhesion structures characteristic of monocytic cells that are implicated in the migration and invasion of cells with the capacity to cross and invade boundaries^19^. They are characterized by a distinctive organization: they are formed by a core of F-actin surrounded by a circular array of integrins and integrin-associated proteins^19^. Chemotactic factors trigger podosome initiation and subsequent binding of integrins to their ligands, including fibronectin and ICAM-1, promoting increased size and maturation of podosomes and adhesion stability^20^. Podosomes are also sites of matrix metalloprotease accumulation with high extracellular matrix (ECM) degradation activity^21^.

The adhesion of monocytes to the inflamed endothelium involves, among other molecules, the integrin family of transmembrane proteins. By binding to the ECM, integrins propagate signals from outside the cell to the cytoskeleton through several intracellular signaling pathways^22^. Integrin-linked kinase (ILK) is a key component of the integrin signaling complex that functions as both an intracellular scaffold molecule and a kinase regulating proliferation, migration, and cell survival^23^. ILK-associated signaling proteins include protein kinase B (PKB/AKT), glycogen synthase kinase 3*β* (GSK-3*β*), and mitogen-activated protein kinases^24^. In vivo and in vitro models show that ILK plays a critical role in vascular vessel integrity, is essential for ECM and endothelial cell interactions and regulates the recruitment and adhesion of both endothelial progenitor cells and human mononuclear cells to the endothelium^25–28^. Our group has demonstrated the role of ILK in the regulation of endothelial nitric oxide production and vasomotor tone^29^. We have also observed that uremic serum (from patients with advanced CKD), *p*-cresol (pc) and IS induce the activation of the ILK protein in endothelial cells with a protective role against oxidative stress and decrease the proliferation and apoptosis of these cells^24^. Interestingly, we have also shown that ILK is required for the accumulation of integrin-associated intracellular proteins in podosome rings downstream of initiation of actin core formation, which determines the adhesive and invasive properties of immature dendritic cells across ECM-based barriers^30^. Therefore, the present study aimed to investigate whether podosome formation is involved in pc and IS toxin-induced monocyte adhesion and whether it may underlie monocyte extravasation. In addition, we analyzed the role of ILK protein in the podosome formation process in this CKD pathophysiological context.

## Material and methods

### Cell culture and treatments

The human leukemic monocyte line THP-1, derived from the peripheral blood of a 1-year-old male with acute monocytic leukemia, was maintained in RPMI culture medium supplemented with 20 mM L-glutamine, antibiotics (penicillin, 100 U ml^−1^; streptomycin, 100 mg ml^−1^), and 10% fetal bovine serum. The cell suspension culture was maintained between 2.5 and 5 × 10^5^ cells ml^−1^. Cells were cultured at 37 °C in a 5% CO_2_ atmosphere. For the experiments, cells were incubated with the different treatments at variable concentrations and times (see figure legends).

The uremic toxins pc, IS, and pCS were tested at concentrations in the uremic range as previously described^24,31^. Briefly, pc was prepared in methanol at a stock concentration of 100 mg ml^−1^, and IS and pCS were prepared in water at a stock concentration of 12.5 mg ml^−1^. The uremic solutes were diluted at least 1:1000 in culture medium to reach mean uremic concentrations for which the final concentration of methanol was <0.1%. The uremic solutes were compared with their respective controls (methanol or water).

### Cell adhesion and podosome formation assays on fibronectin

Freshly prepared 10 μg ml^−1^ fibronectin solution was incubated over 13 mm-diameter sterile glass coverslips in 24-well plates for 1 h at 37 °C before plating cells. THP-1 cells (5 × 10^5^) were plated on the fibronectin-coated coverslips in 500 μl of RPMI per well and treated with pc and IS or 1 ng ml^−1^ transforming growth factor beta 1 (TGF-*β*1) as a positive control. After 24 h of incubation, the cells were washed once with PBS, fixed for 20 min in 4% (w/v) paraformaldehyde/3% (w/v) glucose in PBS, permeabilized for 15 min with 0.05% Triton X-100 in PBS and blocked for 30 min with 3% bovine serum albumin in PBS at room temperature. For localization of filamentous actin, cells were incubated with phalloidin for 45 min at room temperature and washed three times with PBS. The nuclei were stained for 5 min with Hoechst 33342 at room temperature, and the coverslips were washed three times with PBS and mounted onto slides using ProLong^TM^ Gold antifade reagent. The samples were analyzed using a LEICA TCS-SP5 confocal microscope (Leica Microsystems, Wetzlar, Germany). Four sequential confocal optical sections were analyzed for the fibronectin ECM of randomly chosen fields. To calculate the percentage of attached THP-1 cells, the percentage of adhered cells in control conditions was considered to be 100%. The percentage of cells with podosomes was determined by the ratio between the number of cells with podosomes and the total number of cells per field.

### Immunostaining and colocalization assay

For immunostaining against ILK and cortactin or Wiskott-Aldrich syndrome protein (WASP), cells were treated and fixed as described above. For immunostaining against vinculin and WASP-interacting protein (WIP), cells were fixed with 100% ice-cold methanol for 5 min. After blockade, THP-1 cells were stained with primary antibodies diluted in 3% bovine serum albumin in PBS at room temperature for 1 h. After three PBS washes, the samples were incubated with appropriate secondary antibodies diluted in 3% bovine serum albumin in PBS for 1 h at room temperature. The cells were stained with phalloidin and Hoechst 33342, and the coverslips were mounted as described above. The samples were analyzed using a LEICA TCS-SP5 confocal microscope (Leica Microsystems, Wetzlar, Germany). Four sequential confocal optical sections were analyzed for the fibronectin ECM of randomly chosen fields.

### Matrix degradation assay

Fluorescent gelatin was prepared as indicated by the Gelatin Invadopodia Assay protocol. Briefly, 13 mm-diameter sterile glass coverslips in 24-well plates were incubated with poly-L-lysine for 20 min, glutaraldehyde for 15 min and red fluorescent gelatin in darkness for 10 min at room temperature. Coverslips were washed three times with PBS after incubation with each reagent. Then, the coverslips were sterilized in 70% ethanol for 30 min, and residual free aldehydes were quenched in RPMI medium for 30 min at room temperature. THP-1 cells (2.5 × 10^5^) were resuspended in 500 μl of RPMI, seeded onto gelatin-coated coverslips and incubated for 24 h with pc and IS or TGF-*β*1 as a positive control. After incubation, the cells were fixed for 20 min in 4% (w/v) paraformaldehyde/3% (w/v) glucose in PBS, permeabilized for 15 min with 0.05% Triton X-100 in PBS, blocked for 30 min with 3% bovine serum albumin in PBS and stained for 45 min with phalloidin at room temperature. The nuclei were stained for 5 min with Hoechst 33342 at room temperature, and the coverslips were mounted onto slides using ProLong^TM^ Gold antifade reagent. The samples were analyzed using a LEICA TCS-SP5 confocal microscope (Leica Microsystems, Wetzlar, Germany). Four sequential confocal optical sections were analyzed for the gelatin ECM of randomly chosen fields. We scored the percentage of THP-1 cells with an associated subjacent area of gelatin degradation against the total cells per field of view. A degrading cell percentage of 100% was assigned for the control conditions.

Moreover, fibronectin images were converted to binary images in which fibronectin appeared black with white degradation spots. A threshold was applied (keeping the threshold the same for all images), and the ImageJ Analyze Particles tool was used to measure the total degradation area for each image. The same process was carried out using the F-actin channel to measure the total cell area in each image. The degradation area was divided by the total cell area. For each treatment condition, the mean degraded area/cell area (µm^2^) was calculated.

### Cell migration assay

The assays were carried out by using an 8 μm pore filter (Transwell, 24-well plate) for the indicated conditions^30^. The lower chambers of the Transwells were filled with 500 μl of RPMI with MCP-1 (0.1 μg ml^−1^) used as a chemoattractant. THP-1 cells (5 × 10^5^) were loaded in 500 μl of RPMI with the corresponding pc and IS treatments in the upper chamber of the filter of each well. After 24 h of incubation, the cells in the upper chamber and the cells that had migrated to the bottom chamber were resuspended and counted using an automatic cell counter. To determine the percentage of THP-1 cells that migrated across the filters toward the MCP-1-containing chamber, the lower cell and upper cell ratios were calculated. One hundred percent migration was assigned to the control condition.

### Conditional ILK-knockdown mice and study design

The animals were housed in a pathogen-free and temperature-controlled room (22 ± 2 °C). Food and water were available ad libitum. The inducible ILK-knockdown mouse (cKD-ILK) model has been explained in prior publications (see Supplemental Material)^32^. After the ILK depletion period, wild-type (WT) and cKD-ILK mice were sacrificed, and blood was collected in tubes with 0.1% EDTA as an anticoagulant. Erythrocytes were lysed in BC FACS^TM^ Lysing Solution, and leukocytes were counted using an automatic cell counter. Uncleaved ILK mRNA levels were measured in leukocytes by RT–qPCR to verify that ILK depletion also occurred in these cells^32^. The primers GGGCTCTTGTGAGCTTCTGT and GAGTGGTCCCCTTCCAGAAT, designed to recognize the cDNA sequence between exons within floxed areas 6 and 7^32^, were used. In cKD-ILK mice, the genetic depletion of ILK resulted in a significant decrease (range of 55–75%) in uncleaved ILK mRNA levels in leukocytes compared to the levels in WT leukocytes. Half of the leukocytes obtained were used to study adhesion and podosome formation by immunostaining assay. Freshly prepared 10 μg ml^−1^ fibronectin and 1 ng ml^−1^ TGF-*β*1 solution were incubated over coverslips in 8-well chambers for 1 h at 37 °C before plating cells. A total of 5 × 10^4^ leukocytes from each animal were plated on the fibronectin- and TGF-*β*1**-**coated coverslips and treated with high concentrations of pc and IS. After overnight incubation, the cells were processed as described above for cell adhesion assays, podosome formation assays, and immunostaining. The samples were analyzed using a LEICA TCS-SP5 confocal microscope (Leica Microsystems, Wetzlar, Germany). Four sequential confocal optical sections were analyzed for the fibronectin ECM of randomly chosen fields. For calculation of the percentage of attached leukocytes, the percentage of cells adhered under WT control conditions was considered 100%. The percentage of cells with podosomes was determined by the ratio between the number of cells with podosomes (WASP-positive cells) and the total number of cells per field. The other half of the leukocytes obtained were used to study the phosphorylation of GSK-3β and AKT proteins by flow cytometry. The leukocytes were treated with high concentrations of pc and IS for 1 h. After incubation, Cell Signaling Buffer Set A was used according to the manufacturer’s guidelines. At the end of the procedure, the cells were stained with GSK-3β pS9-APC and AKT pS473-PE antibodies and incubated for 30 min at room temperature. The fluorescence intensity of the cells was evaluated by flow cytometry in a MACSQuant® Analyzer 10 Flow Cytometer (Miltenyi Biotec) with 640 nm and 488 nm argon lasers. GSK-3β pS9 and AKT pS473 phosphorylation was estimated using the median fluorescence intensity (MFI) of the cell population. We considered the WT control condition as 100%.

### Statistical analysis

The results are presented as the mean ± standard error of the mean (SEM). All experiments were repeated at least three times (the number of experiments is provided in the legends of the figures). Normality of the value distributions was assessed by the Kolmogorov–Smirnov test. In those cases, two-way ANOVA was the chosen test. In those comparisons including two experimental conditions in the same experiment (for instance, treatments and times) that were standard or nested (when paired criteria were used), two-way ANOVA followed by a post-hoc analysis was the selected statistical method. Otherwise, Kruskal–Wallis (nonpaired, more than two groups) or Friedman (paired, more than two groups) tests, followed by Mann–Whitney or Wilcoxon posttests (with the Bonferroni correction), were used. GraphPad Prism version 5.00 for MacIntosh (San Diego, CA) was used for the analyses. A *P-*value of *<* 0.05 was considered to indicate statistical significance.

## Results

### pc and IS increase ILK activity in THP-1 cells

**Fig. 1:**
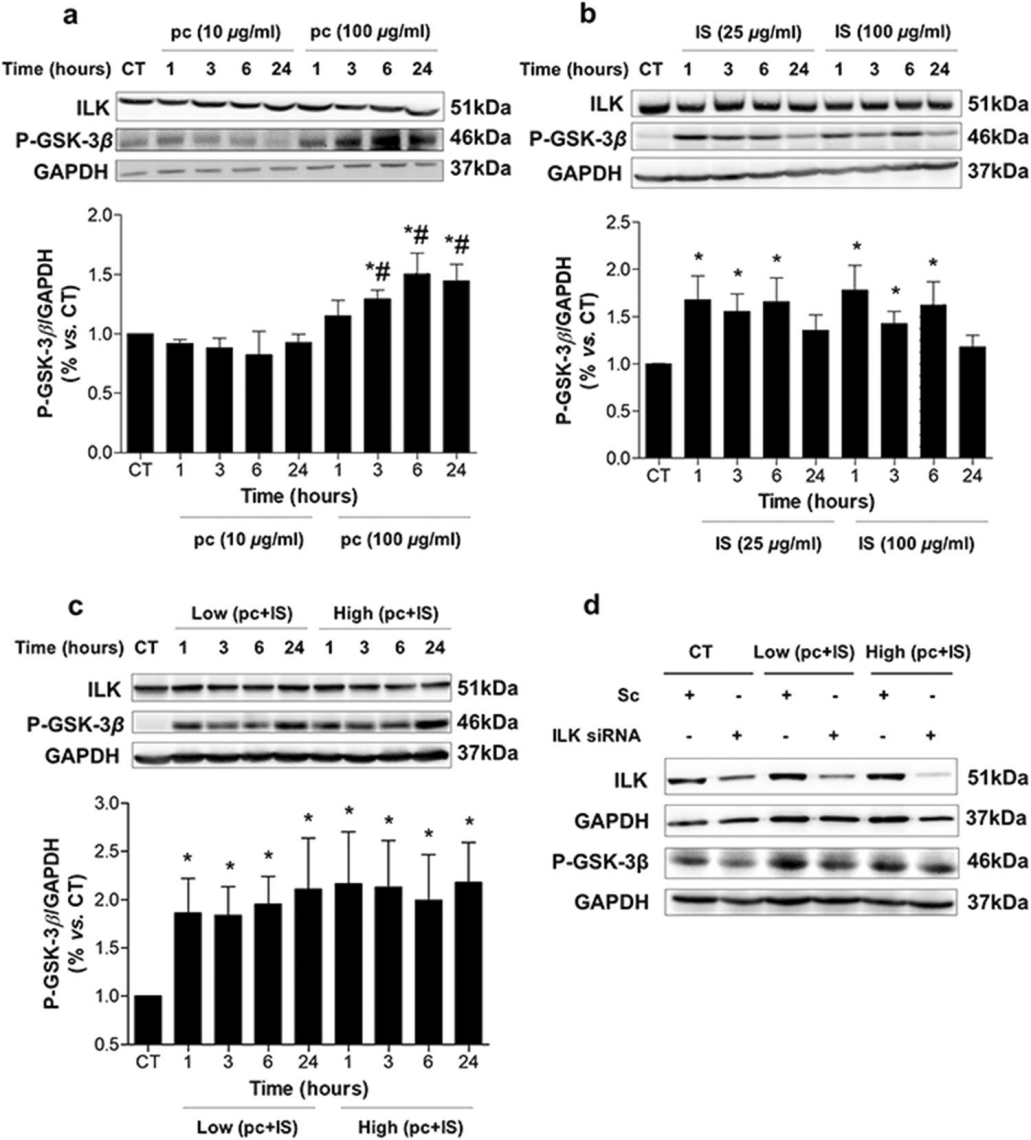
*p*-Cresol (pc) and indoxyl sulfate (IS) upregulate ILK activity in THP-1 cells. THP-1 cells were incubated with **a** pc, **b** IS, or **c** low concentrations of pc plus IS or high concentrations of pc plus IS for different times. **d** THP-1 cells were transfected with scrambled RNA (Sc) or were depleted of ILK with specific siRNA and treated as in **c** for 24 h. Representative western blots of GSK-3*β* phosphorylated on the serine-9 residue (P-GSK-3*β*) or ILK are shown. GAPDH was used as the endogenous control. The bars represent the normalized densitometric values of the blots against the endogenous control values. All values are presented as the mean ± SEM from 4 independent experiments. *P < 0.05 vs. untreated control (CT); ^#^*P* < 0.05 vs. pc (10 *μ*g ml^−1^).

The effects of pc (a surrogate of the main in vivo metabolite, pCS) and IS on ILK activity and expression were tested in THP-1 cells. As shown in Fig. 1a, b, exposure of cells to these toxins generated different stimulation patterns of ILK kinase activity, as determined by an increase in the phosphorylation levels of the ILK downstream effector GSK-3β at serine-9. pc induced a significant increase in GSK-3β phosphorylation after 3 h of incubation at 100 µg/mL compared to that in nonexposed cells (Fig. 1a). Exposure to IS at 25 and 100 µg/mL for 1, 3, and 6 h also increased the levels of phosphorylated GSK-3β compared to the levels in control cells (Fig. 1b). Interestingly, cells treated with a combination of both toxins at previously used low or high concentrations induced both rapid and sustained increases in GSK-3β phosphorylation (Fig. 1c). In addition, we confirmed that the increased phosphorylation of GSK-3β was ILK-dependent because it was reversed when ILK was knocked down by ILK siRNA, as measured by western blotting (Fig. 1d). In contrast, incubation with pc and IS did not affect either ILK cellular content (Fig. 1a-c) or mRNA expression (Supplementary Fig. 1). Under the experimental conditions selected, pc and IS did not induce any significant toxicity in THP-1 cells (Supplementary Fig. 2a, b). Based on these results, the combination of both toxins was selected for the following experiments. Additional experiments were performed with an equivalent dose of pCS (22.6 or 226 μg/mL) instead of pc or a mixture of 100 μg/mL IS plus 226 μg/mL pCS instead of 100 μg/mL IS plus 100 μg/mL pc to analyze whether pc and pCS have similar effects on ILK activity and expression. The results presented in Supplementary Fig. 3a, b show that treatment of cells with pCS or IS plus pCS did not increase ILK expression and increased GSK-3β phosphorylation levels in the same way as treatment of cells with pc or IS plus pc. pCS did not induce any significant toxicity in THP-1 cells (Supplementary Fig. 2c).

### pc and IS induce monocyte adhesion, podosome formation, ECM degradation, and cell migration in vitro

**Fig. 2:**
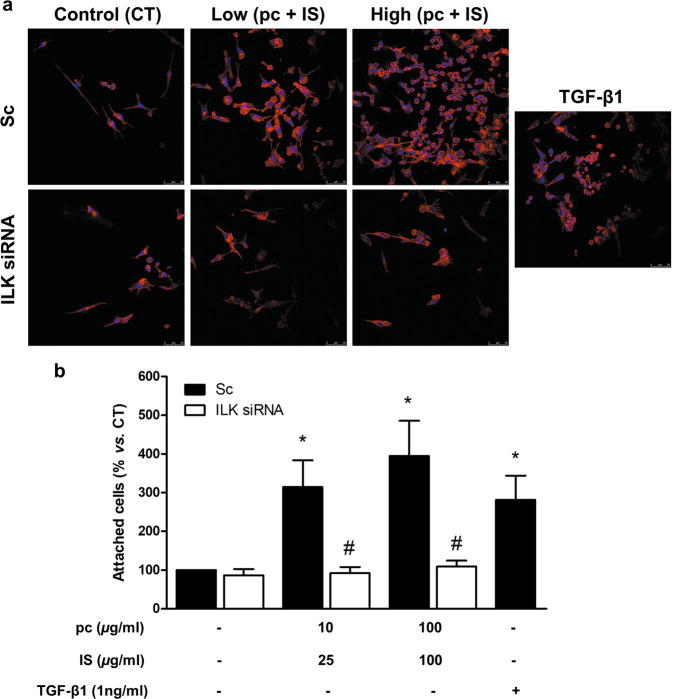
*p*-Cresol (pc) and indoxyl sulfate (IS) induce THP-1 cell adhesion to a fibronectin matrix. THP-1 cells were transfected with scrambled RNA (Sc) as a control (CT) (upper microphotographs, **b** black bars) or were depleted of ILK with specific siRNA (lower microphotographs, **b** white bars). Afterward, the cells were seeded on fibronectin-coated coverslips and incubated with low concentrations of pc plus IS or high concentrations of pc plus IS for 24 h. **a** Adhesion of THP-1 cells stained with phalloidin (red) and Hoechst 33342 (blue) to the fibronectin matrix was determined by fluorescence confocal microscopy. The results of a representative experiment are shown. Scale bar, 50 µm. **b** Bar graphs indicating the average percentages of attached THP-1 cells treated as in **a**. The results are expressed as a percentage of the number of untreated CT cells. All values are presented as the mean ± SEM from 4 independent experiments. *P < 0.05 vs. CT; ^#^*P* < 0.05 vs. Sc. TGF-*β*1 was used as a positive control.

**Fig. 3:**
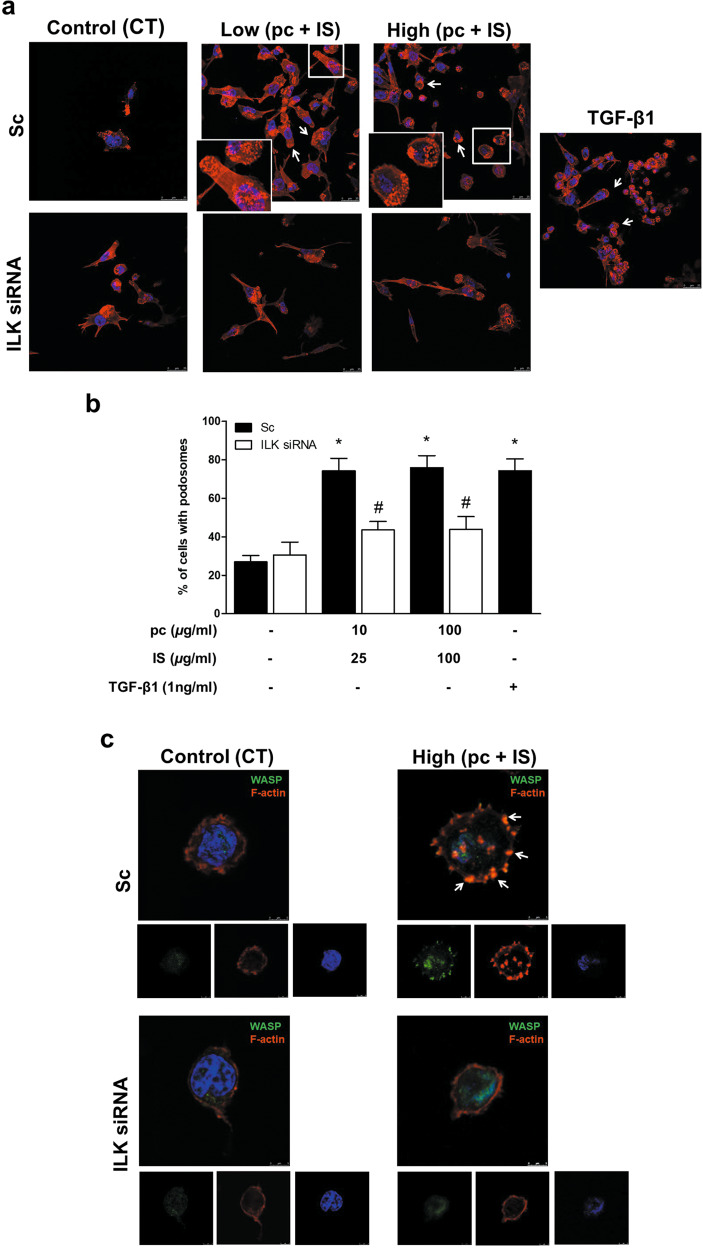
*p*-Cresol (pc) and indoxyl sulfate (IS) induce THP-1 cell podosome formation in a fibronectin matrix. THP-1 cells were transfected with scrambled RNA (Sc) as a control (CT) (**a**, **c** upper microphotographs, **b** black bars) or were depleted of ILK with specific siRNA (**a**, **c** lower microphotographs, **b** white bars). Afterward, the cells were seeded on fibronectin-coated coverslips and incubated with low or high concentrations of pc plus IS (**a**, **b**) or high concentrations of pc plus IS (**c**) for 24 h. **a,** **c** Podosome formation of THP-1 cells stained with phalloidin (red) and Hoechst 33342 (blue) (**a**) or phalloidin (red), WASP (green) and Hoechst 33342 (blue) (**c**) was determined by fluorescence confocal microscopy. A representative experiment is shown. Scale bar, 25 or 5 µm, respectively. Magnifications of the boxed area are shown at the bottom left. **b** Bar graphs showing the mean of the percentage of cells with podosomes per field of view for cells treated as in **a**. All values are presented as the mean ± SEM from 4 independent experiments. *P < 0.05 vs. untreated CT; ^#^*P* < 0.05 vs. Sc. TGF-*β*1 was used as a positive control.

**Fig. 4:**
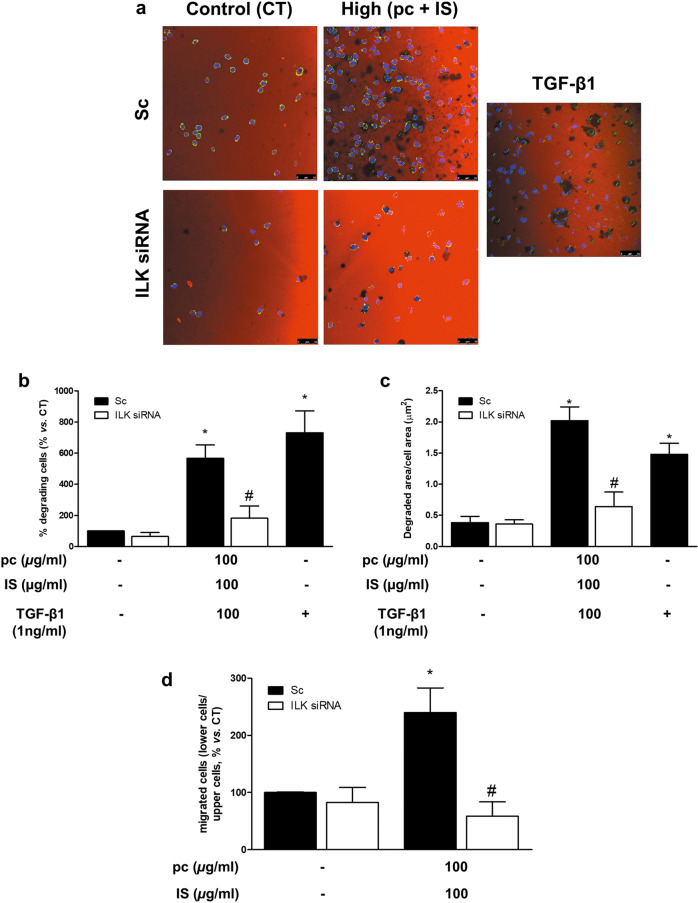
*p*-Cresol (pc) and indoxyl sulfate (IS) induce THP-1 cell matrix degradation and cell migration. THP-1 cells were transfected with scrambled RNA (Sc) as a control (CT) (upper microphotographs, **b**–**d** black bars) or were depleted of ILK with specific siRNA (lower microphotographs, **b**–**d** white bars). **a**–**c** Afterward, the cells were seeded on TRITC-gelatin-coated coverslips and incubated with high concentrations of pc plus IS for 24 h. **a** Confocal micrographs showing the distribution of TRITC gelatin (red) and THP-1 cells stained with phalloidin (green) and Hoechst 33342 (blue). The results of a representative experiment are shown. Scale bar, 50 µm. **b**, **c** Bar graphs indicating the average percentage of THP-1 cells with an associated subjacent area of gelatin degradation (**b**) or the total degraded area divided by the total cell area (µm^2^) (**c**) per field of view for cells treated as in **a**. **d** Afterward, the cells were loaded in the upper chamber of the filter and incubated with high concentrations of pc plus IS for 24 h. Cell migration was determined by Transwell migration assay. The bar graphs indicate the average percentage of THP-1 cells that migrated across the filter toward MCP-1 cells treated as in **a**. **b,** **c** The results are expressed as a percentage of the number of untreated CT cells. All values are presented as the mean ± SEM from 4 or 6 independent experiments. *P < 0.05 vs. CT; ^#^*P* < 0.05 vs. Sc. TGF-*β*1 was used as a positive control. MCP-1 was used as a chemoattractant.

**Fig. 5:**
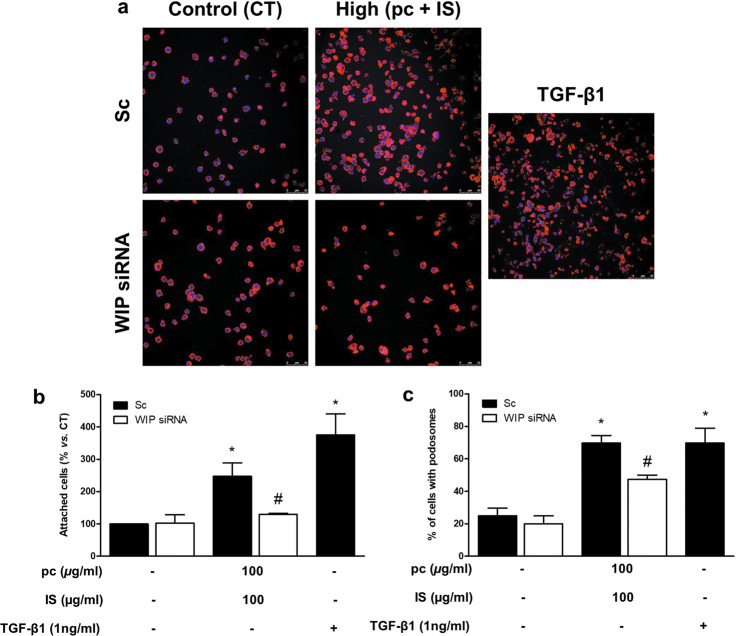
Podosome-specific WIP protein depletion impairs *p*-cresol (pc)- and indoxyl sulfate (IS)-induced cell adhesion to a fibronectin matrix. THP-1 cells were transfected with scrambled RNA (Sc) as a control (CT) (upper microphotographs, **b,** **c** black bars) or were depleted of WIP with specific siRNA (lower microphotographs, **b,** **c** white bars). Afterward, the cells were incubated with high concentrations of pc plus IS for 24 h. **a** Adhesion of THP-1 cells stained with phalloidin (red) and Hoechst 33342 (blue) to the fibronectin matrix was determined by fluorescence confocal microscopy. The results of a representative experiment are shown. Scale bar, 50 µm. **b** Bar graphs indicating the average percentage of attached THP-1 cells treated as in **a**. **c** Bar graphs showing the mean percentage of cells with podosomes per field of view for cells treated as in **a**. The results are expressed as a percentage of the number of untreated CT cells. All values are presented as the mean ± SEM from 4 independent experiments. *P < 0.05 vs. CT; ^#^*P* < 0.05 vs. Sc. TGF-*β*1 was used as a positive control.

To investigate the effects of pc and IS on different monocyte properties involved in vascular damage, THP-1 cells were exposed to a mixture of pc plus IS for 24 h and then seeded on fibronectin-coated culture plates. The mixture of pc plus IS induced significant increases in THP-1 cell adhesion (Fig. 2a, b), the percentage of cells that formed podosomes (Fig. 3a, b), ECM degradation (Fig. 4a-c) and monocyte migration (Fig. 4d) compared to the levels in the untreated control cells. In the cases of adhesion, podosome formation, and ECM degradation, the pc plus IS mixture-dependent monocyte changes were comparable to those induced by TGF-*β*1 (Figs. 2a, b, 3a-c and 4a-c). Furthermore, we confirmed the localization of specific WASP in podosomes together with actin to clearly identify and characterize these structures (Fig. 3c). Additional experiments performed with a mixture of 100 μg/mL IS plus an equivalent dose of pCS (226 μg/mL) instead of pc indicated that treatment of cells with IS plus pCS induced significant increases in THP-1 cell adhesion (Supplementary Fig. 4a, b) and the percentage of cells that formed podosomes (Supplementary Fig. 5a-c) in the same way as treatment of cells with IS plus pc. To confirm whether cell adhesion induced by the uremic toxins was specifically dependent on podosome formation, we depleted the expression WIP, a protein implicated in initiating podosome formation^19^. WIP depletion completely blocked the increased monocyte adhesion in uremic toxin-treated cells (Fig. 5a, b) and significantly reduced podosome formation (Fig. 5c).

### ILK is implicated in podosome-mediated cell adhesion, ECM degradation, and migration of monocytes under in vitro stimulation with pc and IS

**Fig. 6:**
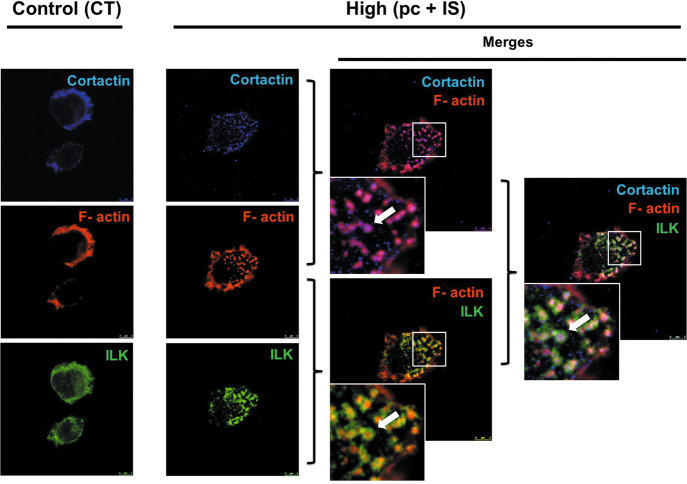
ILK is localized in the podosome rings of THP-1 cells induced by *p*-cresol (pc) and indoxyl sulfate (IS). THP-1 cells were seeded on fibronectin-coated coverslips and incubated with high concentrations of pc plus IS for 24 h. Confocal micrograph showing the distribution of ILK (green) in the podosome ring and colocalization of phalloidin-stained F-actin (red) and cortactin (blue) in the podosome core in THP-1 cells. Scale bar, 5 µm. Magnifications of the boxed area are shown at the bottom left. The experiment was repeated five times.

To analyze the role of ILK in podosome formation, we first explored whether ILK localizes in podosomes under pc and IS treatment, as previously described in dendritic cells^30^. THP-1 cells seeded on fibronectin were exposed to uremic toxins and were simultaneously stained for F-actin, cortactin (podosome-specific) and ILK. F-actin colocalized with cortactin in podosome cores, while ILK was located in the rings of podosomes in the presence of pc and IS (Fig. 6). Then, we confirmed the requirement of ILK for pc- and IS-induced monocyte adhesion and podosome formation by knocking down ILK with a specific small interfering RNA (siRNA). Under this condition, the increased THP-1 cell adhesion to fibronectin (Fig. 2a, b) and podosome formation (Fig. 3a-c) induced by the toxins were completely abolished. The increased ECM degradation capacity of THP-1 cells induced by pc and IS (Fig. 4a-c), as well as the increased chemotaxis of these cells toward MCP-1 (Fig. 4d), were also dependent on ILK, as they were completely abolished when ILK was knocked down with ILK siRNA. Taken together, the present results suggest that ILK participates in different stages of the monocyte transmigration process by regulating the remodeling of F-actin and podosome functionality.

Next, to try to determine putative mediators that activate ILK in the presence of pc and IS, we evaluated reactive oxygen species (ROS) generation and PI3K as possible upstream candidates. Regarding ROS production, we found a significant increase in hydrogen peroxide (H_2_O_2_) accumulation in cells exposed to both the low and high concentrations of combined pc and IS (Supplementary Fig. 6a). Nevertheless, the catalase-dependent decrease in intracellular H_2_O_2_ did not reduce pc- and IS -induced ILK activity (Supplementary Fig. 6b). On the other hand, PI3K activity, measured as phosphorylation of AKT on threonine-308^33^, did not change in the presence of high concentrations of pc and IS (Supplementary Fig. 6c). Therefore, the modulation of ILK activity by these toxins does not seem to depend on ROS production or PI3K activation.

**Fig. 7:**
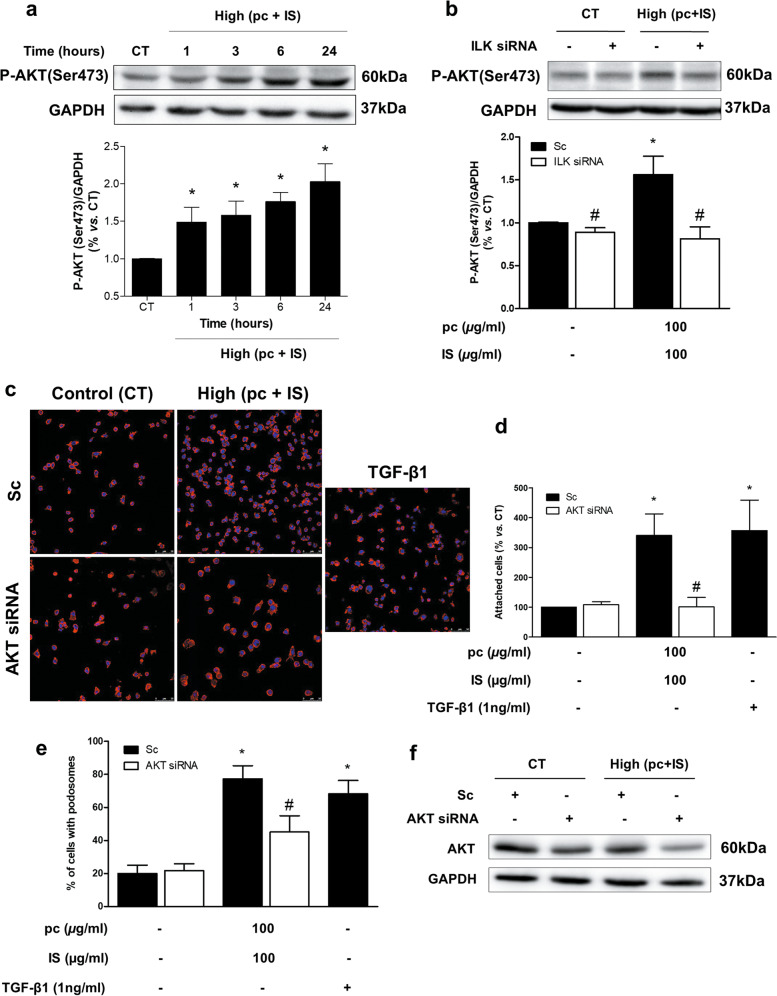
Molecular mechanism downstream of ILK activation. **a** THP-1 cells were incubated with high concentrations of *p*-cresol (pc) plus indoxyl sulfate (IS) for different times. **b** THP-1 cells were transfected with scrambled RNA (Sc) as a control (CT) (black bars) or were depleted of ILK with specific siRNA (white bars). Afterward, the cells were incubated with high concentrations of pc plus IS for 3 h. **a,** **b** Representative Western blots of AKT phosphorylated on the serine-473 residue (P-AKT) are shown. GAPDH was used as the endogenous control. The bars represent the normalized densitometric values of the blots against the endogenous control values. **c**–**f** THP-1 cells were transfected with scrambled RNA (Sc) as a control (CT) (**c** upper microphotographs, **d,** **e** black bars) or were depleted of AKT with specific siRNA (**c** lower microphotographs, **d,** **e** white bars). Afterward, the cells were seeded on fibronectin-coated coverslips and incubated with high concentrations of pc plus IS. **c** Adhesion of THP-1 cells stained with phalloidin (red) and Hoechst 33342 (blue) to the fibronectin matrix was determined by fluorescence confocal microscopy. The results of a representative experiment are shown. Scale bar, 50 µm. **d** Bar graphs indicating the average percentage of attached THP-1 cells treated as in **c**. **e** Bar graphs showing the mean percentage of cells with podosomes per field of view for cells treated as in **c**. The results are expressed as a percentage of the number of untreated CT cells. All values are presented as the mean ± SEM from 3, 4, or 5 independent experiments. *P < 0.05 vs. CT; ^#^*P* < 0.05 vs. Sc. TGF-*β*1 was used as a positive control. **f** Total AKT expression was measured by western blot analysis (AKT). GAPDH was used as the endogenous control.

We also sought to better understand the signaling pathways downstream of the increased ILK activity that were involved in podosome formation. ILK directly activates AKT through phosphorylation on serine-473^34^. As shown in Fig. 7a, a significant increase in AKT phosphorylation at serine-473 was observed after pc and IS toxin incubation. Treatment with IS plus pCS also had similar effects (Supplementary Fig. 7). ILK depletion completely blocked the increase in AKT phosphorylation (Fig. 7b). The involvement of AKT both in cell adhesion (Fig. 7c, d) and in podosome formation induced by pc and IS (Fig. 7e) was demonstrated by silencing AKT in these cells (Fig. 7f), upon which significant suppression of these processes was observed.

### ILK is involved in pc- and IS-enhanced adhesion of leukocytes in mice

**Fig. 8:**
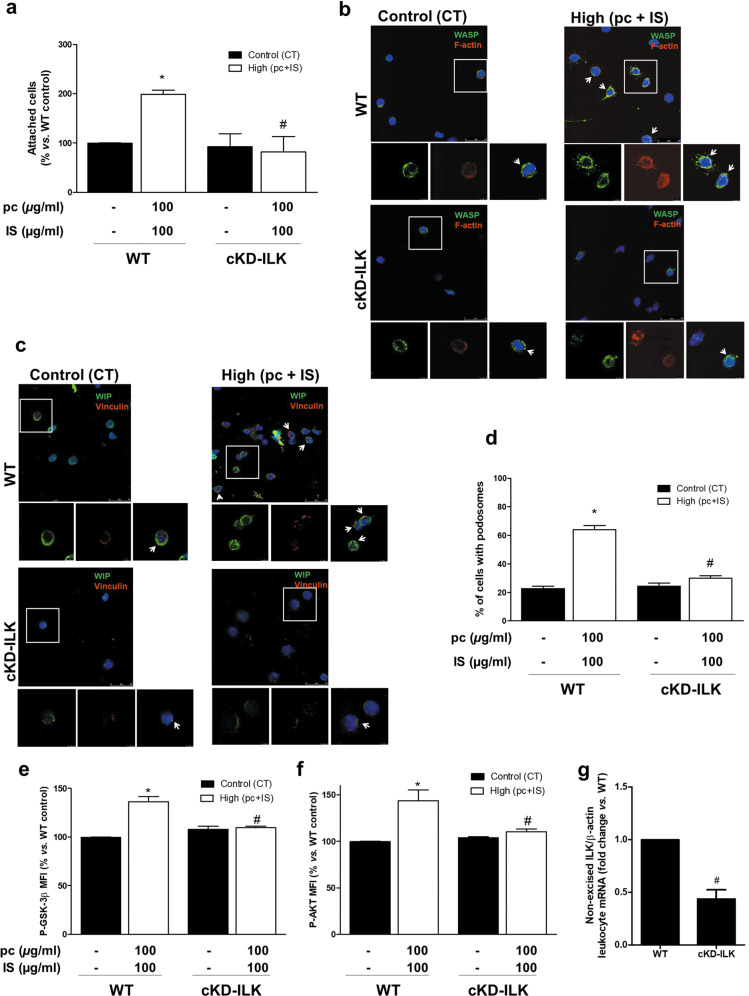
ILK depletion prevents ex vivo increases in podosome formation and adhesion to a fibronectin matrix in mouse leukocytes and the molecular mechanism downstream of ILK activation induced by *p*-cresol (pc) plus indoxyl sulfate (IS) treatment. CRE-LOX mice were injected with tamoxifen (ILK conditional-knockdown [cKD-ILK] mice) or vehicle (wild-type [WT] mice) to induce ILK deletion. Leukocytes were obtained, seeded on fibronectin-coated coverslips, and incubated with high concentrations of pc plus IS for 24 h. **a** Bar graphs indicating the average percentage of leukocytes attached to the fibronectin matrix as determined by fluorescence confocal microscopy. **b, c** The podosome formation of leukocytes stained with phalloidin (red) and a WASP antibody (green) (**b**) or vinculin (red) and WIP (green) antibodies (**c**) as well as Hoechst 33342 (blue) was determined by fluorescence confocal microscopy. The results of a representative experiment are shown. Magnifications of the boxed area are shown at the bottom. Scale bars: 25 and 5 μm. **d** Bar graphs indicating the mean percentage of cells with podosomes per field of view for cells treated as described above. **e**, **f** Median fluorescence intensity (MFI) of GSK-3β pS9 (**e**) and AKT pS473 (**f**) in the leukocyte cell population as analyzed by flow cytometry. The results are expressed as a percentage of the WT control (untreated). **g** Uncleaved ILK mRNA expression in leukocytes was quantified by RT–qPCR. The relative fold changes in mRNA content vs. those in the WT group after normalization to total β-actin content (the endogenous control) are presented. The values are presented as the mean ± SEM from 3 or 5 independent experiments. *P < 0.05 vs. WT control; ^#^*P* < 0.05 vs. (pc + IS) WT.

To investigate whether this response was also present in primary leukocytes, cell adhesion to fibronectin was assayed with leukocytes from control and cKD-ILK mice^32^ incubated ex vivo with pc and IS. The mixture of pc and IS-induced significant increases in control mouse leukocyte adhesion (Fig. 8a) and podosome formation (Fig. 8b-d) that were not observed in leukocytes from cKD-ILK animals (Fig. 8a-d). Finally, we confirmed that pc plus IS induced ILK activation, as determined by increased P-GSK-3β (serine-9) and P-AKT (serine-473) levels in leukocytes, which was prevented by ILK depletion (Fig. 8e, f). Treatment with IS plus pCS also had similar effects (Supplementary Fig. 8a-e). We confirmed that in cKD-ILK mice, transgenic depletion of ILK resulted in a significant decrease in uncleaved ILK mRNA levels in leukocytes compared to the levels in WT mice (Fig. 8g).

## Discussion

The present results strongly support the role of podosome formation in the transmigration potential of circulating monocytes exposed to several uremic toxins, such as those found in CKD patients. The ILK/AKT pathway appears to be involved in podosome regulation under this condition, with subsequent effects on monocyte cell adhesion, migration, and matrix degradation capacity. Therefore, maintenance of low levels of ILK activity could be a potential therapeutic strategy in the prevention of CVD associated with CKD.

Although many studies have focused on the discovery of factors associated with CKD that can produce CVD, less is known about the influence of uremia on the cellular physiopathological mechanisms involved in the leukocyte–endothelium interaction or the extravasation process, which can lead to the development of cardiovascular damage. Uremia has been linked to increasing leukocyte activity and inflammation, which can in part be attributed to the accumulation of uremic toxins^5,14^. In addition, dysfunction and endothelial damage present in almost all patients with CKD seem to be some of the starting elements in the cascade of events leading to CVD^35^. In particular, the interaction of leukocytes recruited to the vascular lesion with the endothelium determines the development of cardiovascular pathologies^36^. Several uremic toxins induce increases in the levels of adhesion molecules, such as ICAM-1, VCAM-1, and E-selectin and inflammatory and chemoattractant factors, such as TNF-α and MCP-1 in endothelial cells^15,37^, as well as the activation and adhesion of leukocytes to the endothelium^13,14,16,17,38^. Moreover, in vivo or in vitro treatment with IS enhances Mac-1 (known to be a receptor for ICAM-1) cell surface expression in monocytes and THP-1 cells through pathways dependent on p38 MAPK and oxidative stress^39^. Mac-1 expression and ROS production are abnormally high in peripheral blood mononuclear cells from incompletely nephrectomized CKD mice^13^. The present work aimed to explore the mechanisms involved in the process of monocyte extravasation under conditions of exposure to pc and IS uremic toxins and is the first study, to our knowledge, that demonstrates the potential involvement of podosomes, which act as transient adhesive structures that degrade the ECM and facilitate the invasive migration of THP-1 myeloid cells.

We have demonstrated the ability of pc and IS to increase ILK kinase activity without affecting its gene expression. We have confirmed the increased adhesion, podosome formation, degradative ECM ability, and MCP-1-induced migration of THP-1 cells under stimulation with pc and IS. All these effects were abrogated by ILK depletion, demonstrating the critical role of this protein in the pc- and IS-induced changes in monocyte properties. In contrast, in a completely different pathophysiological context, functional assays under physiologically relevant flow conditions showed that overexpression of wild-type ILK in human monocytic cells diminished β1 integrin/VCAM-1-dependent firm adhesion to human endothelial cells, suggesting a negative regulation of adhesion by ILK^40^. However, in this context, the effect was attributed to changes in ILK protein content, whereas our data demonstrated that pc- and IS-induced changes in ILK only increased kinase activity, as previously reported in endothelial cells^24^. The differences between cell types illustrate the complex regulation that may take place in the interaction between circulating macrophages and endothelial cells.

Our group and others have shown an absolute requirement of integrin and actin-based adhesive structures called podosomes for normal migration and chemotactic responses in myeloid cells^19,30^. Actin polymerization and integrin remodeling leading to polarization and podosome initiation in myeloid cells are regulated by WASP and WIP^19,20,41^. We have reported that ILK is required for the accumulation of integrin-associated proteins in podosome rings downstream of WASP-mediated initiation of the actin core^30^. In the present study, we found that the ILK protein is necessary for the formation of podosomes induced by pc and IS in monocytic cells. Podosomes can be distinguished from other focal adhesion complexes by the presence of ‘podosomal markers’, such as the proteins gelsolin, cortactin, dynamin 2, and WASP/WIP^42^. By performing double staining for F-actin and cortactin, we confirmed their colocalization in the podosome core, which is typically used to identify podosomal structures, as well as ILK localization surrounding the podosomal actin-cortactin core, as we have previously described^30^. Furthermore, we also confirmed the localization of WASP in the podosomes and observed that inhibition of podosome formation through WIP depletion completely abrogated monocyte adhesion induced by pc and IS^43^. Taken together, these results demonstrate the key role of ILK in the formation of podosomes induced by pc and IS in monocytic cells.

We also investigated the mechanisms underlying ILK-mediated podosome formation, ECM degradation and cell migration in monocytes stimulated with pc and IS. According to previous results from our group and others, ILK directly activates AKT through phosphorylation on serine-473^24,34,44^. Here, we verified that pc and IS induce AKT phosphorylation in an ILK-dependent manner, since ILK depletion completely blocked this response. Furthermore, the knockdown of AKT with specific siRNA impaired the pc- and IS-dependent increase in podosome formation. These results are consistent with recently published findings establishing that AKT activity (achieved by serine-473 phosphorylation) is specifically required for podosome formation in TGF-*β*-treated THP-1 cells^42^. In this work, p21-activated kinase 4 (PAK4) kinase activity is proposed to intersect with the AKT pathway at the podosome ring:core interface, driving the regulation of macrophage podosome turnover, although this mechanism is not due to direct phosphorylation of AKT by PAK4^42^. Since some observations have raised the possibility that ILK activation by PAK4 might be important in the ILK-mediated signaling network^45^, it is possible that in our pc and IS stimulation context, a PAK4-dependent increase in ILK activity could have underlain the increase in AKT phosphorylation. It remains to be elucidated whether and/or how PAK4 is regulated within this process. Interestingly, we previously established ILK as a key mediator in H_2_O_2_-dependent TGF-*β*1 upregulation in human mesangial cells through a P-AKT (serine-473)-dependent mechanism^44^. Since TGF-*β*1 promotes podosome formation, stabilization, and ECM degradation in THP-1 cells^20,42,46^, and since we observed here that this cytokine induces an effect similar to that of pc and IS, an increase in TGF-*β*1 could be one possible mechanism that explains the increases in observed effects dependent on ILK activity.

Podosomes are sites for ECM degradation involved in the degradation of the basement membrane, which allows cells cross this tissue barrier^46,47^. Podosomes recruit transmembrane MT1-matrix metalloprotease and secreted matrix metalloproteases involved in breaking down ECM proteins^47,48^. Here, we found that the impaired adhesion and formation of podosomes in ILK-depleted cells correlated with a decrease in the degradation of gelatin; thus, we conclude that ILK is essential for podosome functionality to degrade the ECM under pc and IS treatment, as we have previously demonstrated in TGF-*β*-stimulated dendritic cells^30^. In invasive cancer cells, ILK also regulates maturation and matrix degradation mediated through invadopodia, which are structures similar to podosomes^48^. Furthermore, we have shown that the depletion of ILK clearly affects both podosome formation and cell migration induced by pc and IS. This agrees with our previous work in which we observed impaired invasive migration of ILK-cKO dendritic cells across Matrigel^30^. Taken together, these data suggest that ILK plays a prominent and essential role in podosome formation, ECM degradation and migration that might favor monocyte extravasation in uremia.

However, the relevance of the results obtained in vitro must be evaluated considering that we used pc instead of pCS, which is the major pc conjugate present in the plasma of CKD patients^49^. There is some evidence in the literature that the effects of both compounds are not very different. Several studies led us to consider that the in vitro effects could be rather similar. pc, pCS and IS all significantly elevate miR-421 levels and decrease ACE2 transcript levels in THP-1 monocytes, which may contribute to the low expression of the enzyme in leukocytes of CKD patients and to the development of atherosclerotic events^50^. Both IS plus pc and IS plus pCS impair skeletal muscle regeneration by reducing myoblast proliferation and preventing chromosome condensation^31^. Moreover, we and others have found similar effects with pooled uremic serum from CKD patients, stimulated endothelial cells and cells exposed to IS plus pc, which induced loss of human endothelial barrier function^51^, decreased cell proliferation, and increased apoptosis and ROS production^24^. Consistent with the abovementioned findings, we demonstrated that the substitution of pc with an equivalent quantity of pCS in some of the experiments performed did not change the results obtained. Conversely, some works have found that pc and pCS have different effects on several cellular functions. In leukocytes, these toxins have opposite impacts: pCS has a proinflammatory effect on unstimulated leukocytes, while pc inhibits the burst activity of leukocytes after stimulation^14^. In endothelial progenitor cells, only pc disrupts cellular function^52^, and in human vascular smooth muscle cells, both toxins stimulate the production of MCP-1, but only pc acts through the NF-κB p65 pathway^53^. Finally, we cannot ensure that the observed effects in our in vitro and ex vivo models would occur in the same way in CKD patients.

Although THP-1 cells are a well-established model system to study podosomes^42^, we also evaluated the extents of mechanisms reportedly associated with ILK in leukocytes isolated from cKD-ILK mice. These cells from cKD-ILK mice had less adherence to the fibronectin matrix than WT leukocytes under pc and IS treatment. This is in agreement with previous in vivo works demonstrating that administration of IS to rats or mice induces leukocyte adhesion to vessel walls^16–18^. Consistently, in uremic rats, AST-120 (an oral adsorbent used in the clinic to reduce plasma IS levels) has been found to suppress an observed increase in monocyte adhesion^38^. Interestingly, we observed that pc and IS induced podosome-like structures and confirmed the podosome localization of WASP and WIP in blood mononuclear cells of control mice, which was not observed in cells from cKD-ILK animals, suggesting a putative role of ILK in podosome-mediated ex vivo adhesion of cells induced by pc and IS.

In conclusion, our findings indicate that pc and IS treatment enhances the attachment and invasive migration of monocytes through activation of ILK/AKT signaling, leading to podosome-mediated motility and matrix degradation (Supplementary Fig. 9). This suggests that ILK may be involved in the migration of monocytes exposed to pc and IS across ECM barriers, a response that may underlie cardiovascular damage in CKD, which primarily relies on interactions among the endothelium, vascular cells, and monocyte-derived macrophages. Monocytes/macrophages are highly plastic cells that show an ability to modify their initial phenotype when facing environmental modifications, such as those in CKD. These phenomena could have important consequences on the ability of these cells to interact with vascular structures and cause injury. Therefore, ILK could be a potential therapeutic target for the treatment of vascular damage associated with CKD.

## Supplementary information

Supplementary information

## Data Availability

The data that support the findings of this study are available from the corresponding author upon reasonable request.
