# Supplementary material for: Indoxyl sulfate- and P-cresol-induced monocyte adhesion and migration is mediated by integrin-linked kinase-dependent podosome formation
Source: Exp Mol Med. 2022 Mar 4;54(3):226–38. doi: 10.1038/s12276-022-00738-8 (PMC8980039; doi:10.1038/s12276-022-00738-8)
Supplement: Supplementary file 1 — Supplementary information [file 12276_2022_738_MOESM1_ESM.pdf]

SUPPLEMENTARY INFORMATION

INDOXYL SULFATE- AND P-CRESOL-INDUCED MONOCYTE ADHESION AND MIGRATION IS MEDIATED BY INTEGRIN LINKED KINASE-DEPENDENT PODOSOME FORMATION

Authors:

Sofía Campillo[1,3], Lourdes Bohorquez[1,3], Elena Gutiérrez-Calabrés[1,3], Diego García-Ayuso[1,3], Verónica Miguel[4], Mercedes Griera[1,3], Yolanda Calle[6], Sergio de Frutos[1,3], Manuel Rodríguez-Puyol[1,3], Diego Rodríguez-Puyol[2,3,5*], Laura Calleros[1,3*]

Affiliations:

[1]Departments of Systems Biology, Physiology Unit, and [2]Medicine and Medical Specialties from Universidad de Alcalá, [3]Instituto Ramón y Cajal de Investigación Sanitaria (IRYCIS), Fundación Renal Iñigo Álvarez de Toledo (FRIAT) and REDinREN (ISCIII), [4]Program of Physiological and Pathological Processes, Centro de Biología Molecular "Severo Ochoa" (CSIC-UAM), [5]Biomedical Research Foundation and Nephrology Unit from Hospital Universitario Príncipe de Asturias, Madrid, Spain, [6]Department of Life Sciences, University of Roehampton, London, UK.

*These authors shared senior direction of this work.

Running title: Monocyte adhesion in uremia mediated by ILK and podosomes

Corresponding author: Laura Calleros Ph.D.
Department of Systems Biology, Physiology Unit,
Facultad de Medicina, Universidad de Alcalá
Campus Universitario s/n.
Alcalá de Henares 28871, Madrid, Spain
Tel/Fax: +34 918854519/ +34 918854590
E-mail address: laura.calleros@uah.es (L. Calleros).

Supplementary Methods

Cell lines and reagents

The human cell line of leukemic monocytes THP-1 and uremic toxins *p*-cresol (pc) and indoxyl sulphate (IS) were obtained from Sigma Chemical Company (St Louis, MO, USA). *p*-cresyl sulphate (pCS) was from Tokyo Chemical Industry (Tokyo, Japan). RPMI medium and antibiotics were from Life Technologies (Carlsbad, CA, USA) and fetal bovine serum were from Lonza (Basel, Switzerland). Recombinant Human transforming growth factor beta 1 (TGF-β1) Protein was from R&D Systems (Minneapolis, MN, USA). ILK-specific small interfering RNA (siRNA) was from Bionova Científica (Barcelona, Spain), and Silencer™ negative control was from Ambion, Inc. (Austin, TX, USA). Metafectene transfection reagent was from Biontex Laboratories (Munich, Germany). Primary antibodies against ILK, P-GSK-3β(Ser9), P-AKT(Ser473), P-AKT(Thr308) and GAPDH and AKT-specific siRNA was purchased from Cell Signaling Technology (Danvers, MA, USA). WASP and WIP antibodies and WIP-specific siRNA were purchased from Santa Cruz Biotechnology (Santa Cruz, CA, USA). Anti-rabbit secondary antibody and QCM™ Gelatin Invadopodia Assay were from Merck Milipore Corp. (Billerica MA, USA) and anti-mouse secondary antibody was from Dako (Barcelona, Spain). ILK, cortactin and vinculin antibodies were from Abcam (Cambridge, UK). Acrylamide-bisacrylamide was from Hispanlab-Pronadisa (Madrid, Spain). Electrophoresis equipment and polyvinylidene difluoride membrane were from Bio-Rad Laboratories (Richmond, CA, USA). Protease and phosphatase inhibitor cocktails were from Roche Diagnostics S.L. (Barcelona, Spain). Chemiluminescence reagent, Cell Tracker™, Alexa Fluor® 488 goat anti-mouse, Alexa Fluor® 647 goat anti-mouse, ProLong™ Gold antifade reagent, Hoechst 33342, Reverse Transcription– Quantitative Polymerase Chain Reaction (RT-qPCR) products, Trypan Blue, Countess™ Automated Cell Counter and H_2DCFDA probe were from Thermo Fisher Scientific (Waltham, MA, USA). Gelatin was from Condalab (Madrid, Spain). Recombinant Human MCP-1 and BC FACS™ Lysing Solution were from BD Biosciences (San Jose, CA, USA). The FastStart DNA Master SYBR Green I Kit was from Roche (Mannhein, Germany). Cell Signaling Buffer Set A and antibodies against GSK-3β pS9-APC and AKT pS473-PE were from Miltenyi Biotec (Bergisch Gladbach, Germany). Bovine serum albumin, fibronectin, phalloidin, Transwell cell culture plate, tamoxifen, CultureWell™ Chambered Coverglass, MTT reagent, Catalase and the rest of drugs or reagents (unless otherwise indicated) were from Sigma Chemical Company (St. Louis, MO, USA).

siRNA transfection

To deplete expression of ILK, AKT or WIP proteins by specific siRNAs,[1] THP-1 cells were cultured with fetal bovine serum-free medium at a concentration of 2.5 × 10^5 cells ml^{-1}. Cells were transfected in fetal bovine serum-free medium with 10 nM ILK-specific siRNA (which depleted ILK protein levels between 50-60%), 20 nM AKT-specific siRNA (which depleted AKT protein levels between 50-70%), 10 nM WIP-specific siRNA (which depleted WIP mRNA levels between 60-80%), or silencer-negative control (Scrambled RNA) using metafectene transfection reagent. After overnight incubation at 37°C in a 5% CO_2 atmosphere with the RNA complex, cell suspension was centrifugated at 1,500 rpm for 5 min and cultured in medium containing fetal bovine serum for 24 h. Then, cells were treated as indicated. To verify the depletion of WIP mRNA levels, TaqMan gene expression assays were used to quantify WIPF1 (Hs00277097_m1) and β-actin (Hs01060665_g1) by RT-qPCR.

Western blot analysis

After treatments, cells were lysed in buffer (10 mM Tris–HCl pH 7.6, 1 mM EDTA, 1% Triton X-100, 0.1% sodium deoxycholate) containing protease and phosphatase inhibitor cocktails. The resulting solution was spun at 11,000 rpm for 20 min at 4°C. Protein concentration for each sample was determined and equivalent amounts of protein (20-40 µg) were run onto 7.5–9% SDS–polyacrylamide gels under reducing conditions and then transferred onto polyvinylidene difluoride membranes. After membranes were blocked with 3% bovine serum

albumin in TBS-T (50 mM Trizma, pH 7.6, 150 mM NaCl, 0.1% Tween 20) for 1 h at room temperature, the membranes were incubated overnight at 4°C with specific antibodies as previously described.[1] This incubation was followed by a second incubation for 1 h at room temperature with their corresponding HRP-conjugated secondary antibody). Membranes were reblotted with mouse anti-GAPDH antibody to normalize protein levels.[2] Antibodies dilutions ranged from 1:500 to 1:5000. Antibody-bound proteins were detected by chemiluminescence (Amersham Biosciences, Amersham, Little Chalfont, UK). Densitometry analyses were performed using ImageJ software (National Institutes of Health, USA).

Conditional ILK Knockdown Mice and study design

Conditional inactivation of the ILK gene was accomplished by crossing C57Bl/6 mice homozygous for the floxed ILK allele (LOX mice) with homozygous mice carrying a tamoxifen-inducible CreER(T) recombinase gene, which express Cre under the control of the cytomegalovirus promoter (CRE mice). Tamoxifen was dissolved in a corn oil/ethanol (9:1) mixture. Male CRE-LOX mice (8-week-old), heterozygous for both transgenes, were injected intraperitoneally with 1.5 mg of tamoxifen once per day for 5 consecutive days to induce ILK deletion. Control animals were injected with vehicle, a corn oil/ethanol (9:1) mixture. Three weeks after the injections, tail DNA was genotyped by PCR with primers allow to distinguish excised ILK gene (230 bp) or to non-excised ILK (2100 bp): CCAGGTGGCAGAGGTAAGTA and CAAGGAATAAGGTGAGCTTCAGAA.[2] PCR DNA products were then analyzed by 1.5% agarose gel electrophoresis. Tamoxifen-treated CRE-LOX mice displaying successful depletion of ILK were termed cKD-ILK mice, and their control vehicle-treated CRE-LOX mice were termed wild-type (WT).

Reverse Transcription– Quantitative Polymerase Chain Reaction (RT-qPCR)

Total RNA from THP-1 cells was extracted using TRIzol, transcribed to cDNA with a High-Capacity cDNA Reverse Transcription Kit, and 10 ng cDNA were amplified in a 7500 qPCR thermocycler. Primers tandems were used to quantify ILK (5′-GACATTGTCGTGAAGGTGCTGAA-3′ (forward) and 5′-GCACTGGGAGCACATTTGGA-3′ (reverse)) and β-actin (5′-TCACCCACACTGTGCCCATCTACGA-3′ (forward) and 5′-CAGCGGAACCGCTCATTGCCAATGG-3′ (reverse)) with SYBR Green Master Mix. $2^{-\Delta\Delta CT}$ method was used for relative quantification of gene expression.[2]

MTT assay

1×10^4 THP-1 cells were seeded on a 96-well culture plate and incubated with 100 µl RPMI containing different treatments at variable concentrations of pc and IS. After 24 h of incubation, 10 µl of 5 mg ml^{-1} 3-(4,5-dimethylthiazol- 2-yl)-2,5-diphenyl-tetrazolium bromide (MTT) reagent in PBS were added to the culture and cells were incubated (4 h, 37°C).[3] Additional wells were incubated with 100 µl RPMI and 10 µl MTT reagent to give blank measurements. After MTT removal, 100 µl DMSO were added to each well and incubated for 10 min at 37°C. Absorbance at 540 nm was measured on a microplate reader and blank measurements were subtracted. Readings from 4 wells per treatment condition were used to mean absorbance levels. 100% metabolic activity was attributed to the one in control conditions.

Cell viability assay

The effect of pc, pCS and IS on cell viability was determined by Trypan Blue exclusion.[1] Briefly, 5×10^5 THP-1 cells were seeded on six-well culture plate and incubated for 6 or 24 h with the different concentrations of pc, pCS and IS. After incubation, cells were centrifuged, resuspended in 1 ml RPMI and diluted ½ in Trypan Blue. The percentage of cells excluding Trypan Blue was determined using a Countess™ Automated Cell Counter.

Reactive oxygen species (ROS) detection

The effect of pc and IS on ROS production, in particular hydrogen peroxide (H_2O_2), was determined by using the H_2DCFDA probe. Catalase was applied as an antioxidant element to decreased intracellular H_2O_2. Catalase-treated THP-1 cells were pre-incubated overnight at a

concentration of 250/500 units. Cells were incubated with H_2DCFDA (5 μM) for 20 min in the dark and washed to eliminate remains of the probe. 5×10^4 cells were re-suspended in 100 μl RPMI with the different concentrations of pc and IS and seeded on 96-well culture plate. Fluorescence emission measurements were performed in a Victor X4 Multilabel plate reader (Perkin Elmer) using 490/535 nm excitation and emission filters. Measurements was taken at indicated times. Readings from 3 wells per treatment condition were used to mean fluorescence levels. ROS production was estimated using the mean fluorescence intensity of the cell population.

Supplementary Figures and Figure Legends

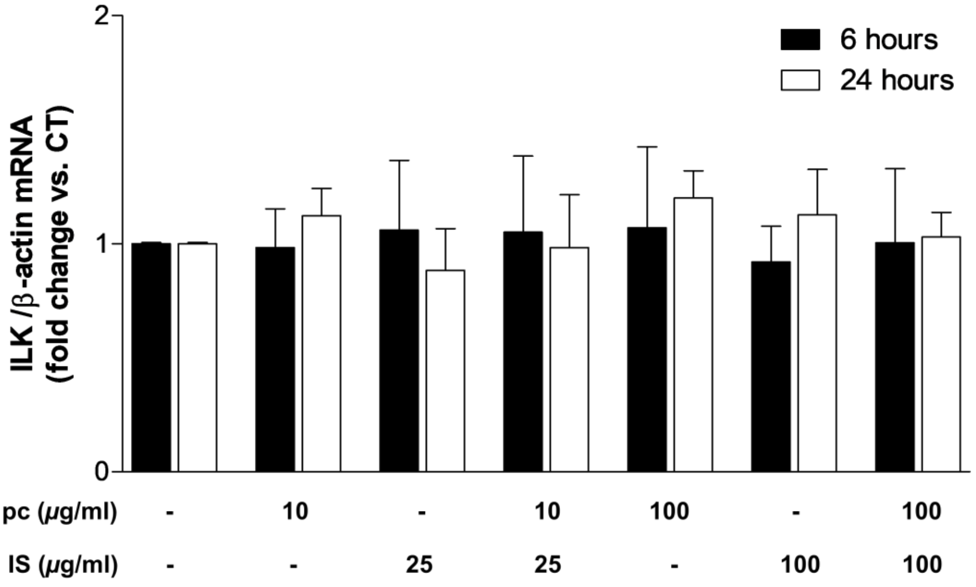

Supplementary Fig. 1 *p*-cresol (pc) and indoxyl sulphate (IS) do not regulate ILK expression in THP-1 cells. THP-1 cells were incubated with pc, IS, a combination of low concentrations of pc plus IS or a combination of high concentrations of pc plus IS for 6 hours (black bars) or 24 hours (white bars). ILK mRNA expression was quantified by RT-qPCR. Relative fold changes in mRNA content vs. untreated control (CT) are represented after the normalization with total β-actin content as the endogenous control. All values are represented as mean ± SEM of 3 independent experiments.

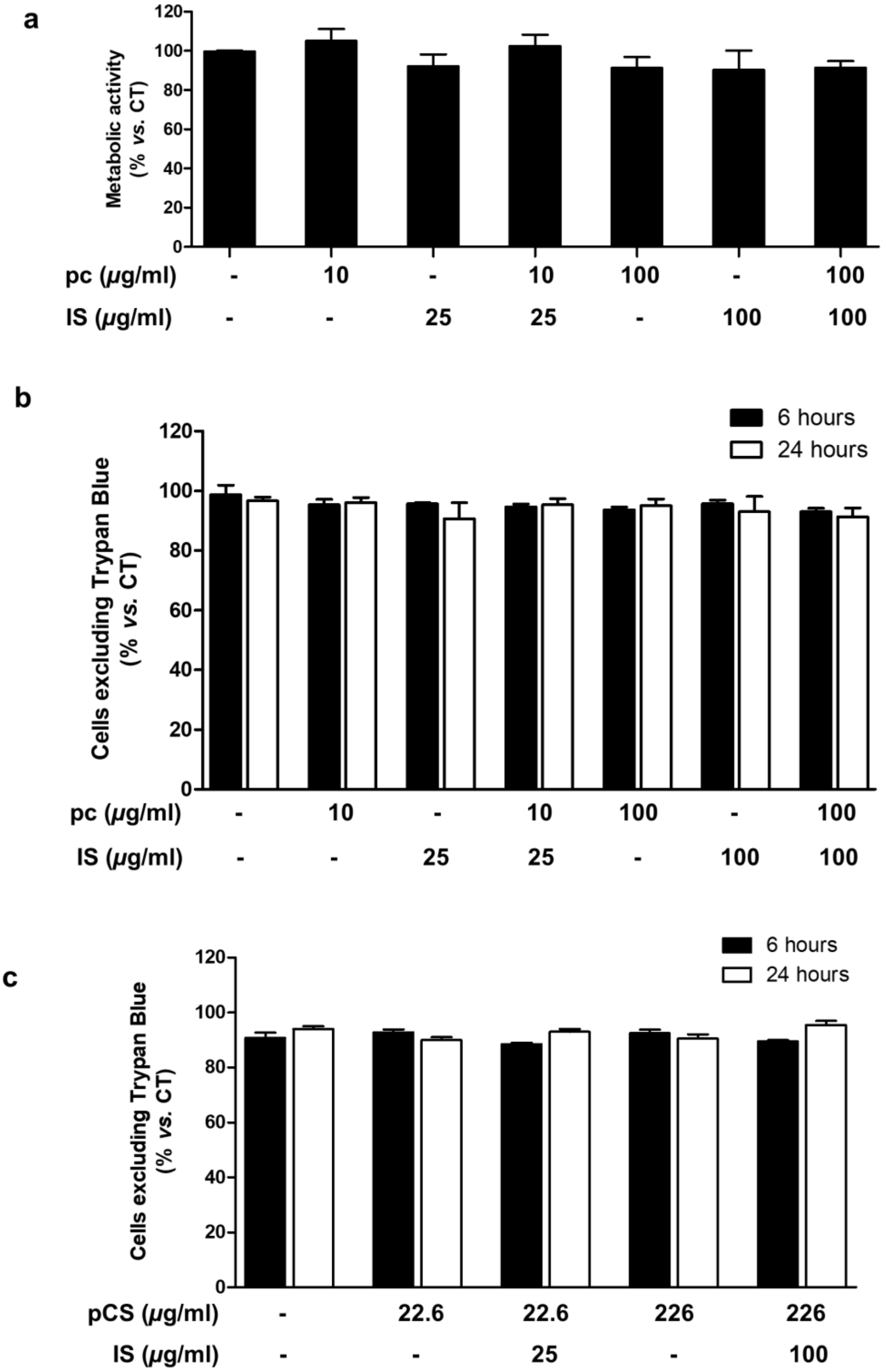

Supplementary Fig. 2 *p*-cresol (pc), *p*-cresyl sulphate (pCS) and indoxyl sulphate (IS) do not affect metabolic activity or viability of THP-1 cells. THP-1 cells were incubated with pc, pCS, IS, a combination of low concentrations of pc plus IS or pCS plus IS, or a combination of high concentrations of pc plus IS or pCS plus IS. (**a**) After incubation for 24 h with the corresponding pc and IS combinations, cell metabolic activity was measured by MTT assay. Results are expressed as a percentage of the number of untreated control (CT) cells. (**b and c**) After incubation for 6 hours (black bars) or 24 hours (white bars) with the corresponding pc, pCS and IS combinations, cell viability was determined by Tripan Blue exclusion. All values are represented as mean ± SEM of 3 independent experiments.

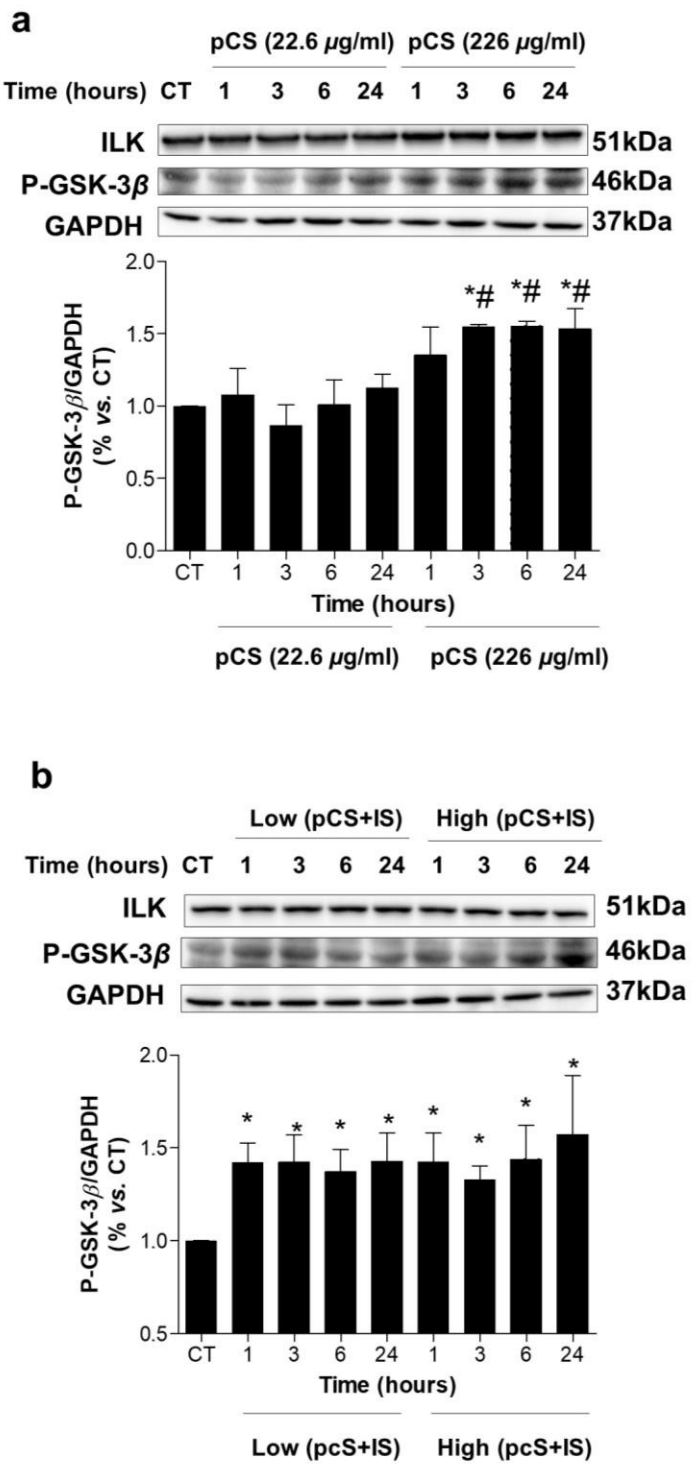

Supplementary Fig. 3 *p*-cresyl sulphate (pCS) alone and plus indoxyl sulphate (IS) up-regulate ILK activity in THP-1 cells. THP-1 cells were incubated with (**a**) pCS or (**b**) a combination of the low or high concentrations of pCS plus IS for different times. Representative Western blots of phosphorylated GSK-3*β* in the serine-9 residue (P-GSK-3*β*) and ILK are shown. GAPDH levels were determined as endogenous control. Bars represent the normalized densitometric analysis of the blots against the endogenous control values. All values are represented as mean ± SEM of 3 independent experiments. *P < 0.05 *vs*. untreated control (CT); #P < 0.05 *vs*. pCS (22.6 μg ml^{-1}).

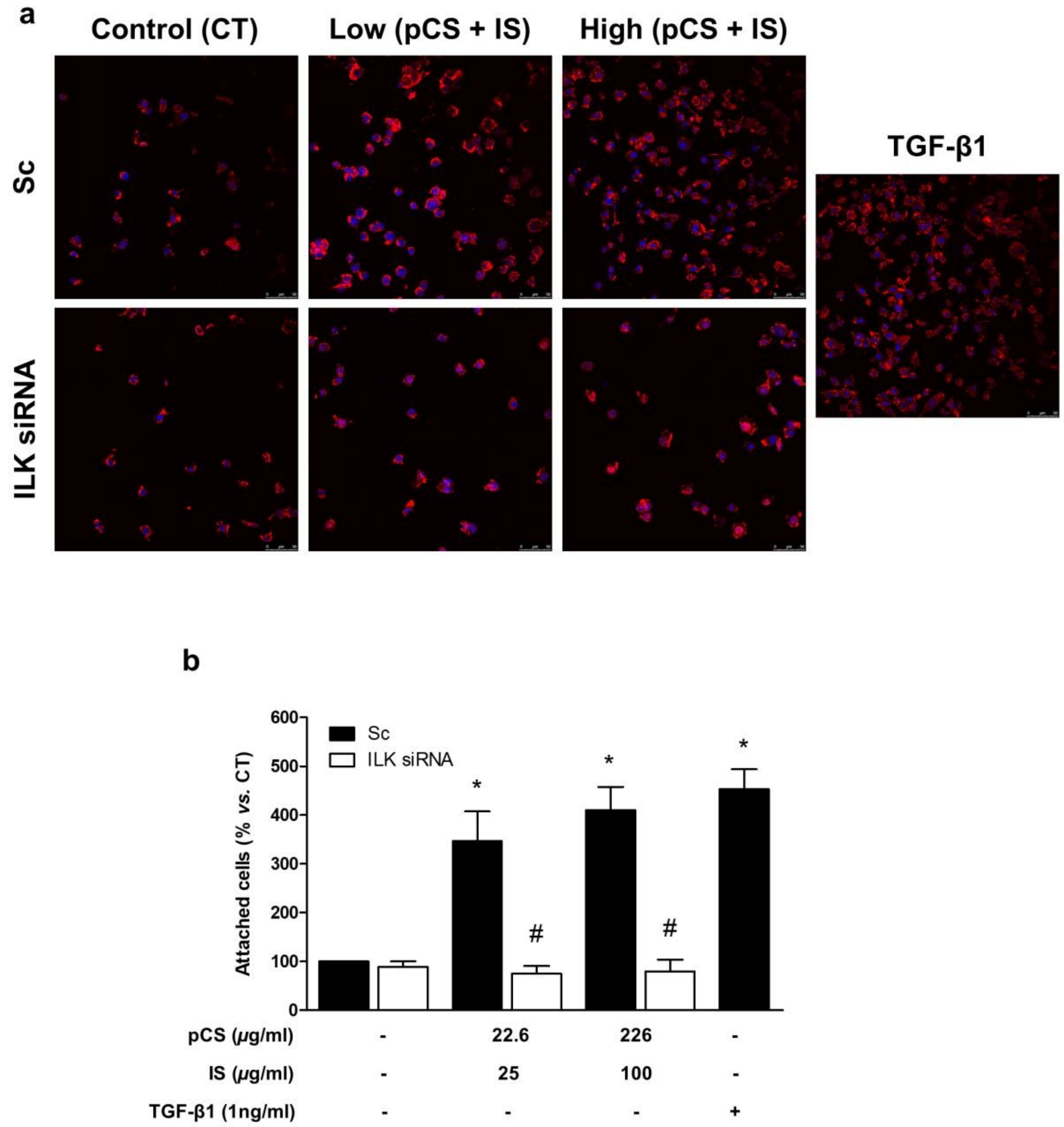

Supplementary Fig. 4 *p*-cresyl sulphate (pCS) plus indoxyl sulphate (IS) induce THP-1 cells adhesion to a fibronectin matrix. THP-1 cells were transfected with scrambled RNA (Sc) as control (CT) (**a** upper microphotograps, **b** black bars) or were depleted of ILK with specific siRNA (**a** lower microphotographs, **b** white bars). Afterwards, cells were seeded on fibronectin-coated coverslips and incubated with a combination of low or high concentrations of pCS plus IS for 24 hours. (**a**) Adhesion of THP-1 cells stained with phalloidin (red) and Hoeschst 33342 (blue) to fibronectin matrix was determined by fluorescence confocal microscopy. A representative experiment is shown. Scale bar, 50 µm. (**b**) Histograms indicate the average percentage of attached THP-1 cells treated as in **a**. Results are expressed as a percentage of the number of untreated CT cells. All values are represented as mean ± SEM of 3 independent experiments. *P < 0.05 *vs*. CT; #P < 0.05 *vs*. Sc. TGF-*β*1 was used as positive control.

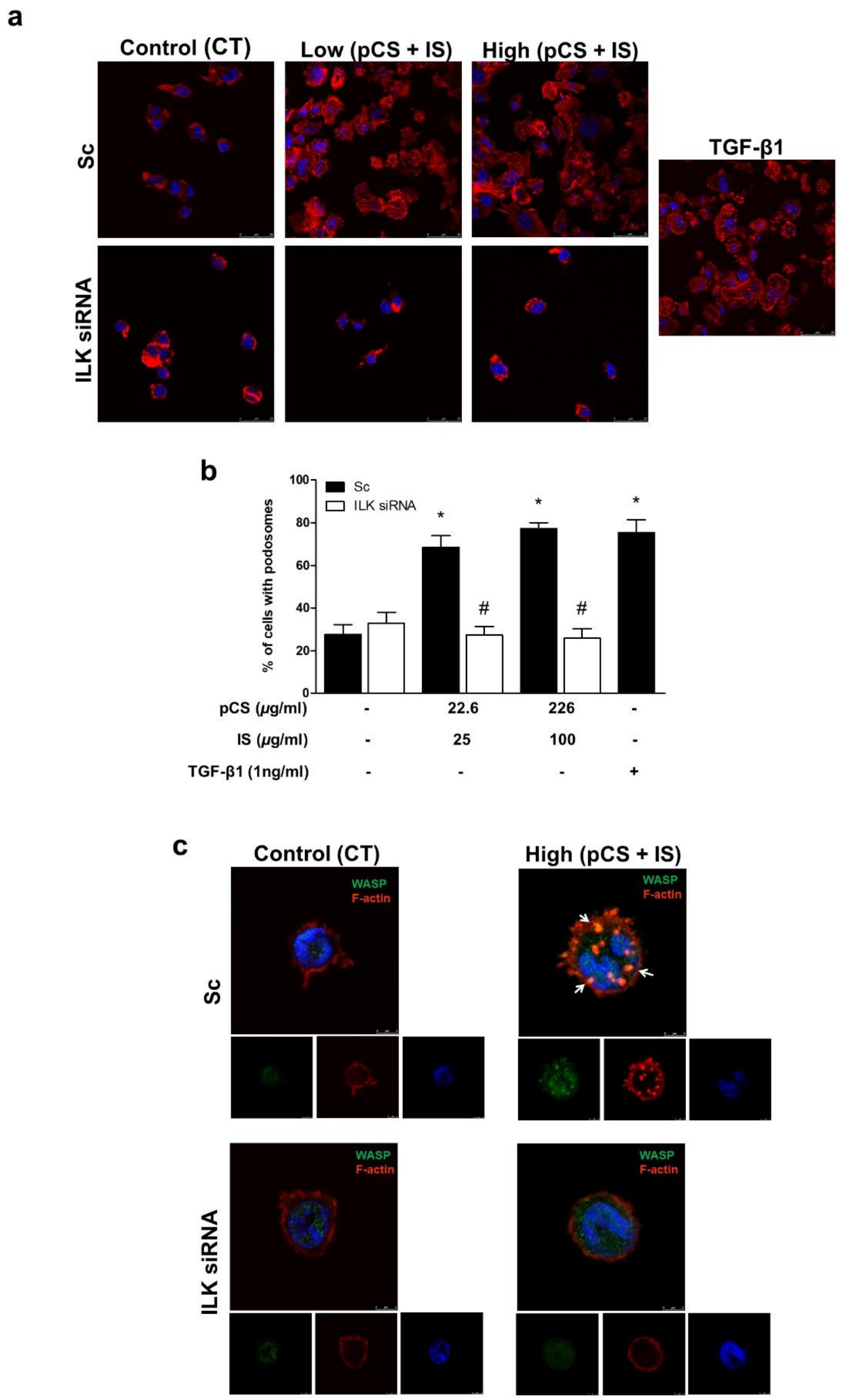

Supplementary Fig. 5 *p*-cresyl sulphate (pCS) plus indoxyl sulphate (IS) induce the THP-1 cells podosome formation in a fibronectin matrix. THP-1 cells were transfected with scrambled RNA (Sc) as control (CT) (**a** and **c** upper microphotographs, **b** black bars) or were depleted of ILK with specific siRNA (**a** and **c** lower microphotographs, **b** white bars). Afterwards, cells were seeded on fibronectin-coated coverslips and incubated with a combination of low or high concentrations of pCS plus IS for 24 hours. (**a** and **c**) Podosome formation of THP-1 cells stained with phalloidin (red) and Hoeschst 33342 (blue) (**a**) or phalloidin (red), WASP (green) and Hoeschst 33342 (blue) (**c**) was determined by fluorescence confocal microscopy. A representative experiment is shown. Scale bar 25 µm or 5 µm respectively. (**b**) Histograms show the mean of the percentage of cells with podosomes per field of view of cells treated as in **a**. All values are represented as mean ± SEM of 3 independent experiments. *P < 0.05 *vs*. untreated CT; #P < 0.05 *vs*. Sc. TGF-*β*1 was used as positive control.

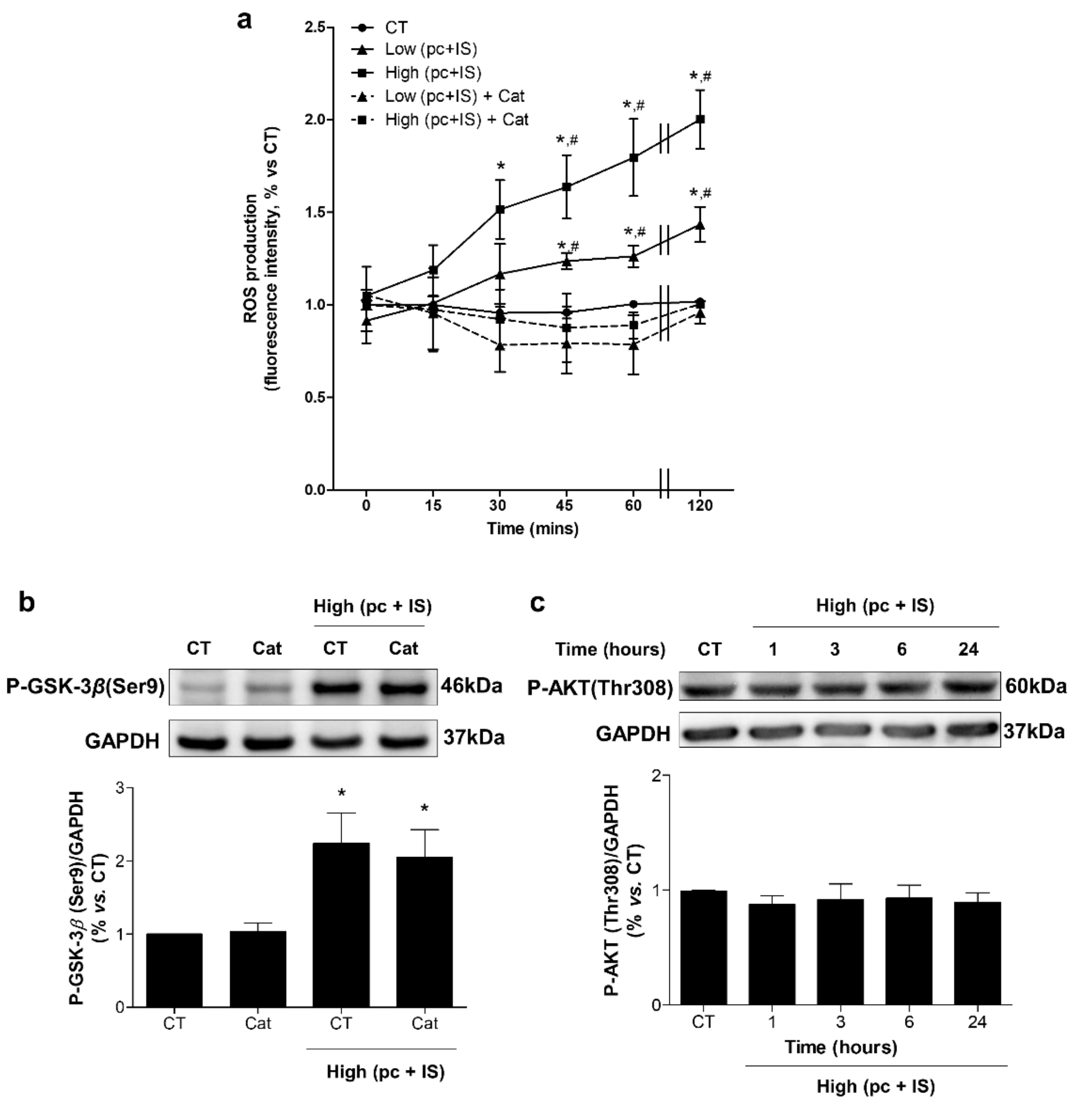

Supplementary Fig. 6 ILK activation by *p*-cresol (pc) and indoxyl sulphate (IS) treatment is neither dependent by ROS production nor by PI3K/AKT pathway in THP-1 cells. (**a** and **b**) THP-1 cells were pre-incubated with catalase (Cat; 250/500 units) as antioxidant overnight (**a** discontinue lines, **b** Cat) or not (**a** continue lines, **b** untreated control (CT)). Afterwards, cells were incubated with a combination of low concentrations of pc (10 *µ*g ml^{-1}) plus IS (25 *µ*g ml^{-1}) (**a** triangles) or a combination of high concentrations of pc plus IS (100 µg ml^{-1}) (**a** squares, **b** High pc + IS). (**a**) CT is represented by circles. Hydrogen peroxide (H_2O_2) production of THP-1 cells was determined by fluorescence emission measurements taken at different times with 490/535 nm excitation and emission filters. (**b**) Representative Western blot of phosphorylated GSK-3*β* in the serine-9 residue (P-GSK-3*β*) is shown. GAPDH levels were determined as endogenous control. Bars represent the normalized densitometric analysis of the blots against the endogenous control values. (**c**) THP-1 cells were incubated with a combination of high concentrations of pc plus IS (100 µg ml^{-1}) for different times. Representative Western blot of phosphorylated AKT in the threonine-308 residue (P-AKT) is shown. GAPDH levels were determined as endogenous control. Bars represent the normalized densitometric analysis of the blots against the endogenous control values. All values are represented as mean ± SEM of 3 or 4 independent experiments. *P < 0.05 vs. CT; #P < 0.05 vs. Cat.

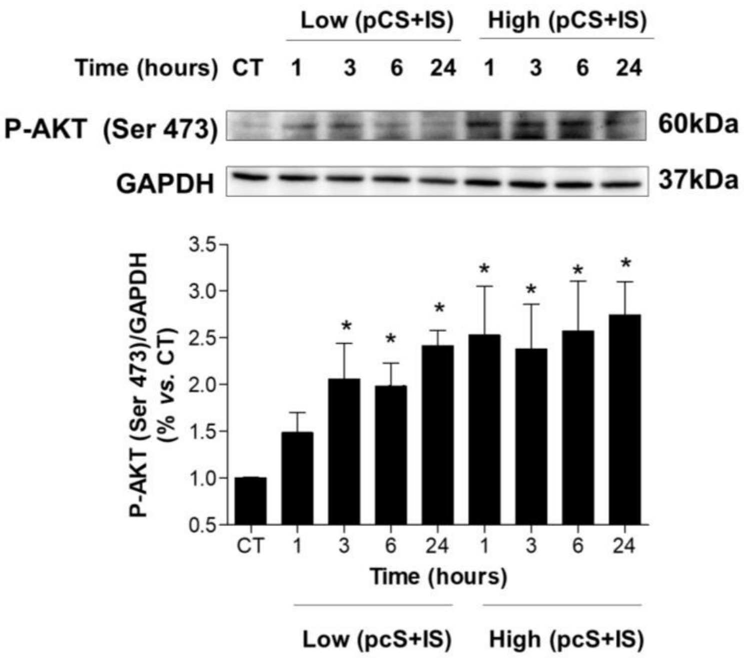

Supplementary Fig. 7 *p*-cresyl sulphate (pCS) plus indoxyl sulphate (IS) increase AKT phosphorylation at serine-473 in THP-1 cells. THP-1 cells were incubated with a combination of the low or high concentrations of pCS plus IS for different times. Representative Western blot of phosphorylated AKT in the serine-473 residue (P-AKT) is shown. GAPDH levels were determined as endogenous control. Bars represent the normalized densitometric analysis of the blots against the endogenous control values. All values are represented as mean ± SEM of 3 independent experiments. *P < 0.05 *vs*. untreated control (CT); #P < 0.05 *vs*. pCS (22.6 μg ml^{-1}).

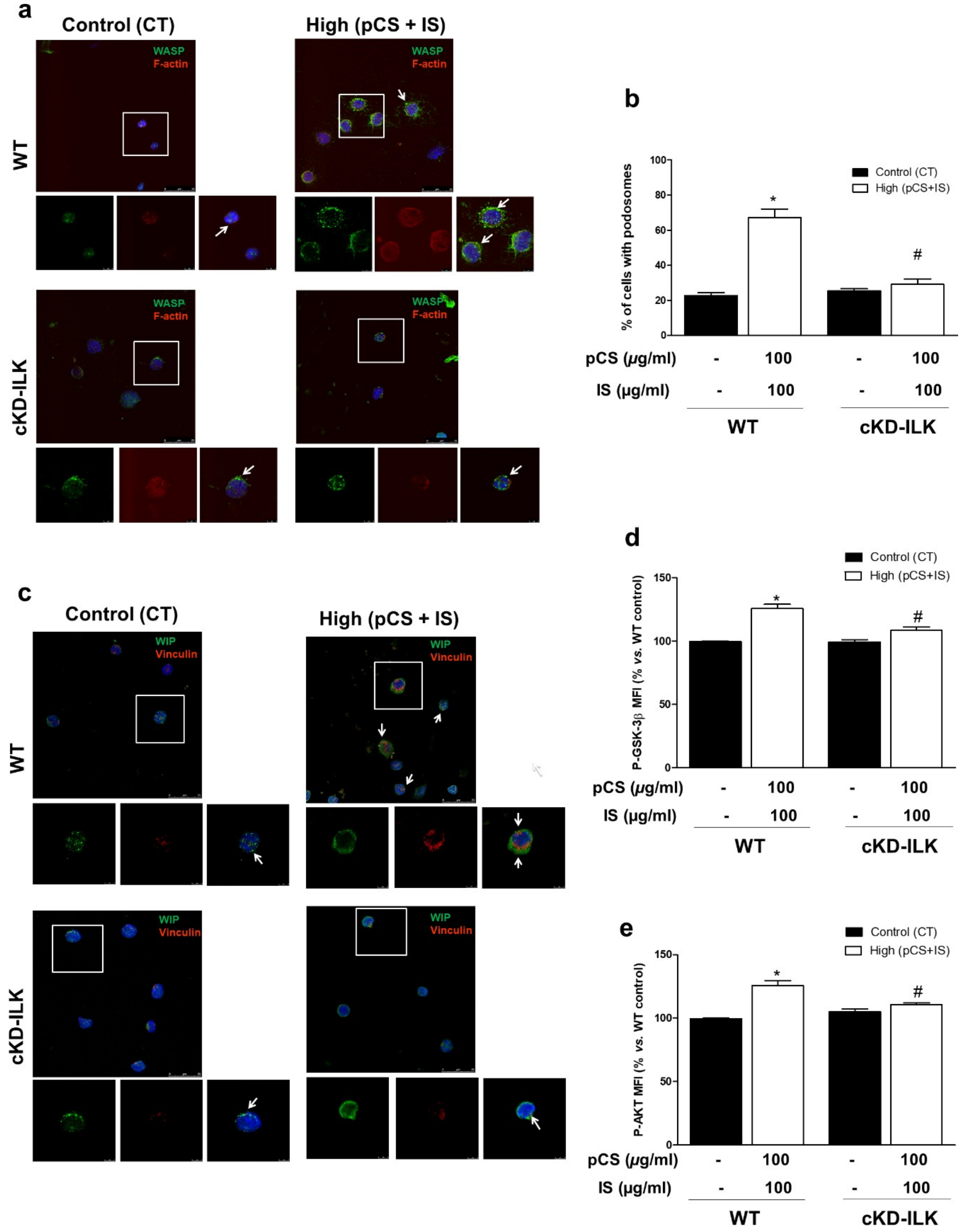

Supplementary Fig. 8 ILK depletion prevents the *ex vivo* mice leukocyte increased podosome formation and the molecular mechanism downstream ILK activation induced by *p*-cresyl sulphate (pCS) plus indoxyl sulphate (IS) treatment. CRE-LOX mice were injected with tamoxifen (ILK conditional-knockdown mice, cKD-ILK) or vehicle (wild-type, WT) to induce ILK deletion. Leukocytes were obtained, seeded on fibronectin-coated coverslips, and incubated with a combination of high concentrations of pCS plus IS for 24 hours. (**a and c**) Podosome formation of leukocytes stained with phalloidin (red) and WASP (green) (**a**) or vinculin (red) and WIP (green) (**c**) and Hoeschst 33342 (blue), was determined by fluorescence confocal microscopy. A representative experiment is shown. Magnifications of the boxed area are shown at the bottom. Scale bars: 25 or 5 µm. (**b**) Histograms indicate the mean of the percentage of cells with podosomes per field of view treated as described above. (**d** and **e**) Median fluorescence intensity (MFI) of GSK-3β pS9 (**d**) and AKT pS473 (**e**) in the leukocyte cell population, analyzed by flow cytometry. Results are expressed as a percentage of WT control (untreated). Values are represented as mean ± SEM of 3 independent experiments. *P < 0.05 vs. WT control; #P < 0.05 vs. (pCS+IS) WT.

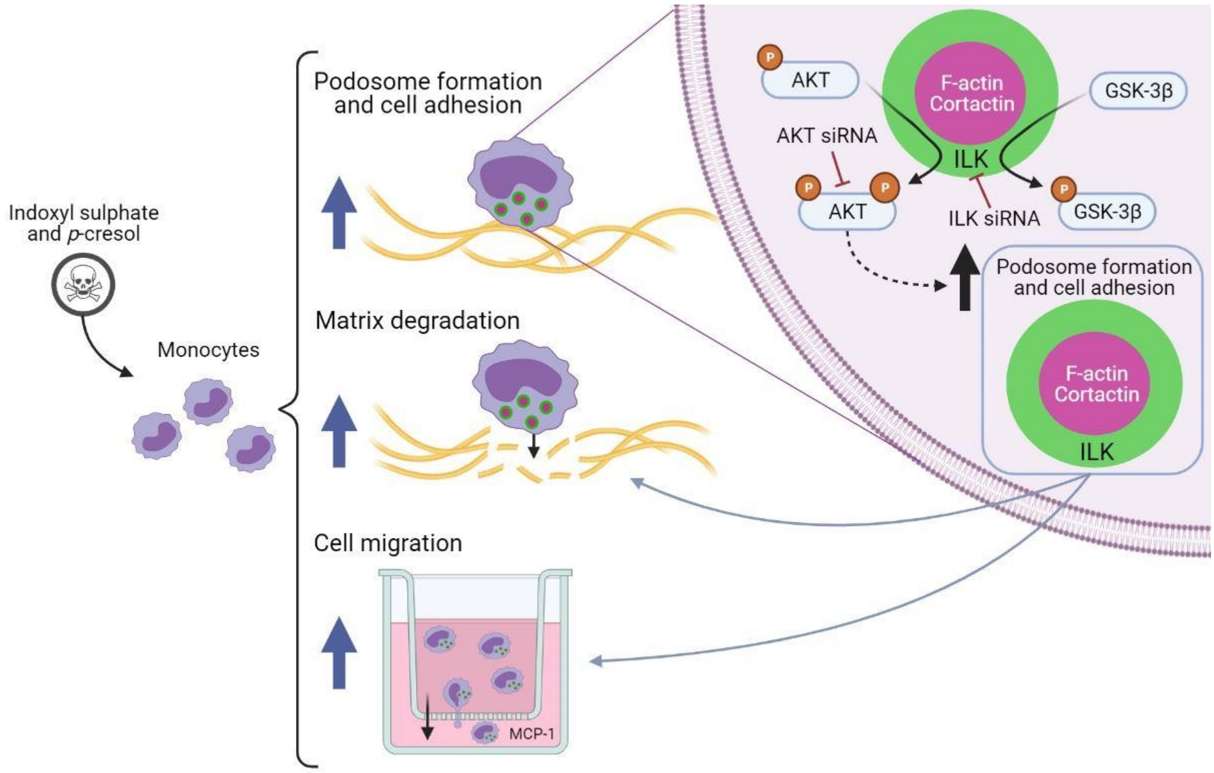

Supplementary Fig. 9 Graphical Abstract

Supplementary References

1. García-Jérez, A. *et al.* Effect of uraemia on endothelial cell damage is mediated by the integrin linked kinase pathway. *J. Physiol.* **593**, 601–618 (2015).

2. de Frutos, S. *et al.* Chronic kidney disease induced by an adenine rich diet upregulates integrin linked kinase (ILK) and its depletion prevents the disease progression. *Biochim. Biophys. Acta - Mol. Basis Dis.* **1865**, 1284–1297 (2019).

3. Foxall, E. *et al.* PAK4 Kinase Activity Plays a Crucial Role in the Podosome Ring of Myeloid Cells. *Cell Rep.* **29**, 3385-3393.e6 (2019).
